# Promoting sexual health in schools: a systematic review of the European evidence

**DOI:** 10.3389/fpubh.2023.1193422

**Published:** 2023-07-04

**Authors:** Ronja Abrams, Johanna Nordmyr, Anna K. Forsman

**Affiliations:** Department of Health Sciences, Faculty of Education and Welfare Studies, Åbo Akademi University, Vaasa, Finland

**Keywords:** sexual health, health promotion interventions, school, Europe, systematic review

## Abstract

**Introduction:**

Sexual ill-health is an urgent public health issue with subsequent social and economic costs. There is, therefore, a need for more effective sexual health promotion interventions in an early stage of life. Previous research has focused on preventive sexual health interventions applying a risk perspective, and the limited and scattered evidence concerning school-based sexual health promotion interventions employing a health-resource perspective has not been compiled and synthesized. Hence, this study aimed to systematically review the current evidence on the effects of sexual health promotion interventions conducted in schools in Europe.

**Method:**

A systematic review based on the JBI and PRISMA standards was performed, encompassing searches in seven databases to identify sexual health promotion interventions conducted in European schools between 2012 and 2022. Data coding was performed according to a predetermined protocol and included information on study characteristics, intervention content, methods, and outcomes relevant to the current review. A narrative synthesis of the included studies was performed, highlighting the collective results.

**Result:**

Seventeen records were included in the review, reporting on 16 individual studies conducted in 7 European countries. Of the 16 included studies, 13 had a quantitative research design, and three had a qualitative design. All three studies with a qualitative research design described positive effects experienced by the participants. Six of thirteen quantitative studies showed statistically significant positive effects on at least one of the outcomes of interest. The outcomes of interest were grouped into five areas, and most studies focused on the area of attitudes toward sexual health.

**Conclusion:**

The findings indicate promising evidence of effect for interventions with a health promotion approach, highlighting the importance of strengthening sexual health resources related to respect, communication skills, attitudes, and other positive psycho-social aspects of sexual health. Most sexual health promotion intervention studies have focused on sexual health resource outcomes connected to attitudes and skills, whereas a comprehensive focus on the multi-dimensional sexual health literacy concept is less common and can be recommended to be included in future intervention research.

## 1. Introduction

Previous research shows that sexual ill-health causes high social and economic costs (1–3). Sexually transmitted infections (STIs) are common globally (1, 4), and the frequency of STI diagnoses has increased in the EU/EEA, especially among young people, even though prevention-focused sexual health education and programs have increased in availability (4). The preventive intervention approach has, in other words, not been sufficient in protecting and promoting the sexual health of the population. There is a need to guide adolescents in how to make informed sexual health choices, as well as to support the development of health resources that promote sexual well-being and resilience in dealing with risks related to sexual health. Sexual health promotion interventions form a key public health strategy to meet these needs (4–6), and more effective, cost-effective as well as engaging sexual health promotion interventions in an early stage of life are warranted (2, 5, 7).

The school context constitutes a critical arena for universal health promotion actions (8), including promoting and supporting pupils’ and students’ health and health literacy (9)—also concerning sexual health and related resources (10). Reaching essentially all children and adolescents, regardless of socio-economic background, schools are a crucial platform for students to learn about sexual health and to promote their capacity to make informed decisions regarding sexual health (11). Sexual health literacy means being able to place health into context, understand what influences health, and then furthermore know how to address the influences for the best possible health outcome for oneself and ones’ family/community (12, 13). Thus, sexual health literacy is a combination of critical skills in various dimensions of sexual health, important to develop throughout life and particularly important in adolescence (14, 15), since this is a period in life when sexual curiosity increases, sexual debut usually occurs, and sexual identity is shaped (16, 17). It is also a period in life when psychological development, social connectedness, and an increasingly independent role in society are critical (18, 19). However, few review studies have focused on the health promotion effect of sexual health literacy in school-based programs for adolescents—although focusing on health literacy as a health promotion action is considered a holistic, sustainable, and cost-effective strategy (10, 20).

Healthy adolescent sexuality development is, in accordance with the definition by WHO (21), more than just the absence of diseases and requires emotional, social, and cognitive skills to enable a sense of well-being in relation to sexuality (22, 23). Furthermore, Kågesten and van Reeuwijk (14) have conceptualized a sexual well-being framework for positive sexual health development in adolescents and argue that there are six key competencies needed: (a) sexual literacy, (b) gender-equitable attitudes, (c) respect for human rights and understanding of consent, (d) critical reflection skills, (e) coping skills and stress management, (f) interpersonal relationship skills. These conceptualizations of sexual health and sexual well-being form the framework for the sexual health promotion perspective in the current study. When a positive sexual health development and a positive understanding of the self in relation to others is promoted in adolescence, it will be beneficial not only in this specific period in life but also in the future life of the adolescents (17, 23).

Nonetheless, existing literature shows that the research has focused on the sexual health of adolescents from a risk and prevention perspective, with sexually transmitted infections (STIs) and unwanted pregnancies in focus in both research and practice (23–25). Consequently, numerous review studies have analyzed the effects of sexual health interventions in schools from a risk-perspective (26–28), showing that a risk approach is neither an optimal nor an effective prevention approach (29, 30). Moreover, the risk approach does not cover the positive sexual health content that adolescents themselves wish to learn more about (17, 25, 31), which is perhaps why another review study found evidence of positive intervention effects when adolescents participate in the planning and implementation of programs (8). The health promotion approach shows promising evidence of effect (2, 5, 32), nonetheless, the health promotion perspective regarding adolescent sexual health is still understudied (5, 14, 23). There is a lack of synthesized evidence on the sexual health resources that adolescents want to learn about that can promote sexual health and well-being, e.g., sexual health literacy (14), self-esteem (6), respect and social skills regarding sexuality (23).

Furthermore, previous review studies covering adolescent sexual health have primarily focused on North America (33) or employed a global perspective (32, 34). Europe differs in many ways from North America, for instance, politically, culturally, and socio-economically which affects education as well as sexual health approaches in schools (35, 36). According to WHO (35), personal growth is generally emphasized in European sexuality education and interventions, while the USA, in contrast, has a more prevention-oriented and problem-solving approach. Studies from the USA have previously reported high rates of teenage pregnancies in comparison with most countries in Europe and other developed countries (36). Perhaps consequently, an abstinence-only focus in sexual health interventions has been dominant in the USA (35, 37). However, abstinence-only programs are proven to be an ineffective sexual health prevention method (35, 38, 39). In Europe, on the other hand, there is a more holistic approach in the interventions, and adolescent sexuality is not seen as a problem but instead a valuable part of a person (35). Previous studies have argued that for sexual health promotion in schools to be effective, socioeconomic as well as political dimensions need to be taken into consideration (9, 40). There are, of course, differences between the European countries—also regarding sexuality education and policies (29, 41). However, the differences between countries are, with a few exceptions, remarkably smaller than in comparison with, for instance, North America (35). Thus, to minimize contextual heterogeneity when considering the evidence of effective sexual health promotion interventions, this review focuses on the European evidence. To the authors’ knowledge, there are no previous European systematic review studies evaluating sexual health-promotion interventions in the school setting focusing primarily on outcomes related to sexual health resources.

### 1.1. Objectives

This review aims to systematically gather and synthesize the current evidence on sexual health promotion interventions in order to assess the evidenced effects of sexual health promotion programs conducted in schools and targeting adolescents in Europe.

## 2. Methods

This systematic review is structured in accordance with the PRISMA (Preferred Reporting Items for Systematic Reviews and Meta-Analysis) (42) guidelines and further follows the guidelines by Joanna Briggs Institute (JBI) for a mixed methods systematic review (43).

### 2.1. Search strategies

Databases were systematically searched using tailored search strategies in April 2022. The following electronic databases were searched: PubMed, CINAHL, ERIC, Education Research Complete, Web of Science, Scopus, and PsycINFO. The terms used in the search strategy can be categorized into population terms (e.g., adolescents, students), geographic terms (e.g., Europe), context terms (e.g., school), program terms (e.g., intervention, action study), and finally outcome terms (e.g., sexual health, sexual well-being, sexual health promotion). Boolean operators and MeSH terms were used as appropriate in the different databases. In addition to the database searches, the reference lists of the included articles and previous review studies were hand-searched with similar inclusion criteria as for this study. See Supplementary file 1 for further details on the applied search strategies.

### 2.2. Inclusion and exclusion criteria

Studies that met the following inclusion criteria were considered eligible: (a) published between 2012 and 2022; (b) conducted in a European country/countries; (c) targeting adolescents (age 12–19); (d) carried out in high school and upper secondary school/vocational school by teachers, health professionals or non-governmental organizations (e) reported on at least one outcome connected to sexual health-promotion and/or positive aspects regarding sexual health. Study designs excluded from this review were cross-sectional-, case, and review studies. Likewise, book chapters, theses, and studies that were not written in English were also excluded. Moreover, studies applying a risk perspective and/or only measuring the risk aspects of sexual health (e.g., knowledge about STIs, and unplanned pregnancy) were excluded. Studies that focused on a specific group of adolescents not representative of the general population (e.g., special education classes) were also excluded.

### 2.3. Study selection and data extraction

**Figure 1:**
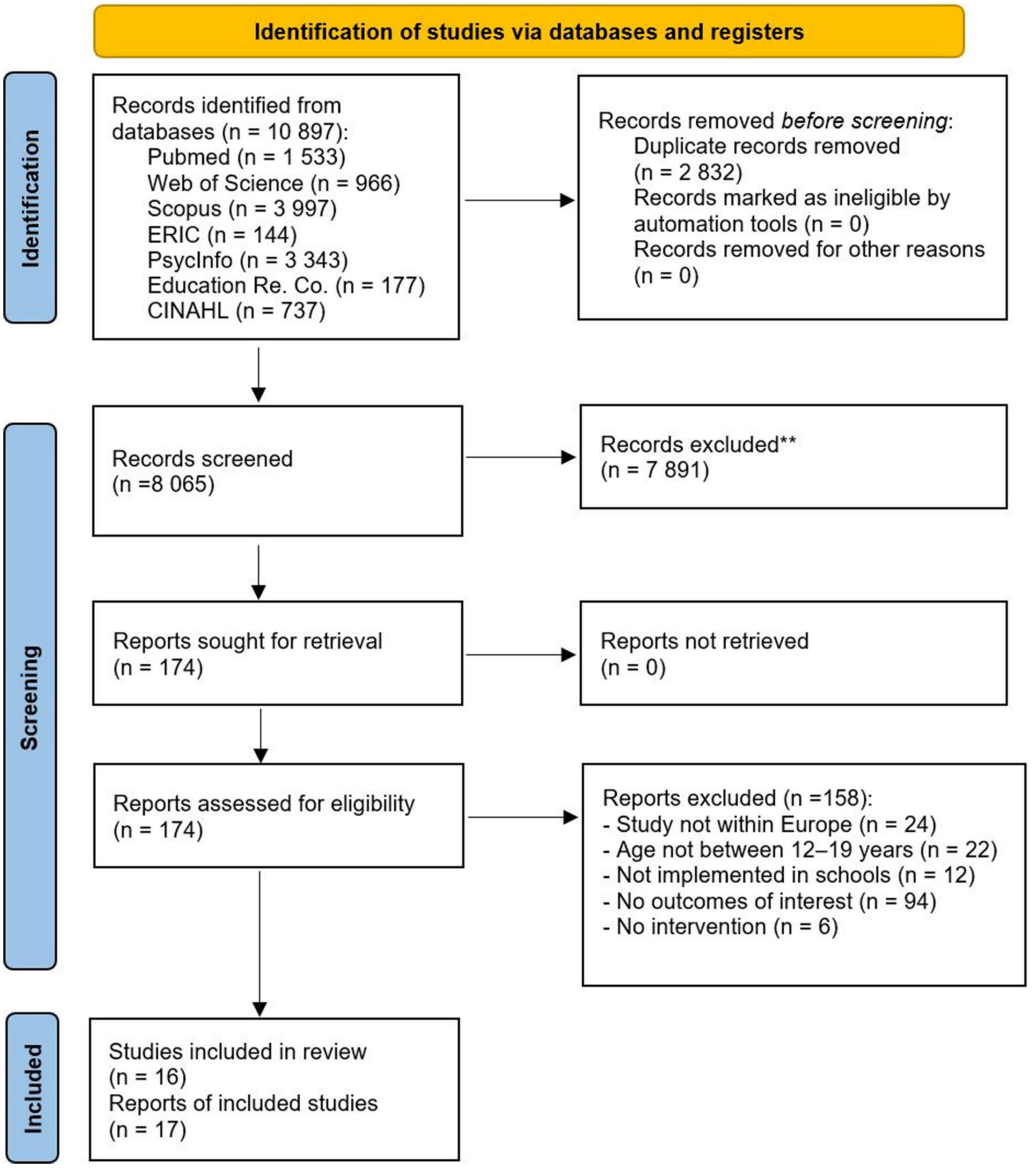
PRISMA flow diagram. From: Page et al. (42).

Article eligibility was initially assessed by one reviewer (RA), who screened the titles and abstracts to exclude duplicates and obviously irrelevant studies. Full texts were read when abstracts met inclusion criteria and when abstract information was insufficient in order to determine eligibility. A second reviewer (JN) assessed the potential eligibility of all the records. Disagreements between reviewers regarding study inclusion were resolved by discussion with reviewers AKF and KG. See the PRISMA flow chart (Figure 1) for detailed information regarding the identification, screening, and record selection process. Initially, 10,897 records were identified, and when duplicates were removed, 8,065 records remained for screening. When titles and abstracts had been screened, 174 records remained for full-text assessment, which after appraisal, led to a list of 45 studies that were assessed and discussed together with the other reviewers (JN, KG, AKF). Studies considered solely to apply a risk perspective were excluded after critical discussions. Thus, finally, 17 records were included in the review, reporting on 16 individual studies. Information from the included records was systematically extracted into a matrix (Supplementary file 2) according to a predetermined protocol to summarize and analyze relevant characteristics. Parallel data extraction was conducted by a second reviewer (JN).

### 2.4. Data synthesis and analyses

Data coding was performed according to a protocol and included study characteristics (e.g., aim, study design), intervention content (e.g., focus and duration), study methods (e.g., sample,), outcomes relevant to the current review (e.g., sexual attitudes, sexual self-esteem) as well as study results. For studies with a quantitative research design, statistical values from the analyses of the intervention effect were extracted from the final point of measurement (e.g., final follow-up measurement). A narrative synthesis of the included studies was performed, highlighting the collective results. See Supplementary file 2 for the extracted data and key information of the included studies.

### 2.5. Risk of bias assessment

The risk of bias of the included intervention studies in relation to study design, conduct, and analysis was assessed and rated according to principles for critical appraisal developed by JBI (44), as well as the corresponding risk of bias checklists developed by the National Institute for Health and Care Excellence, NICE (45, 46). The critical appraisal instruments have been developed to account for variations in research designs and applied methods among studies included in systematic reviews. Moreover, in accordance with the NICE guidelines, each study was awarded a quality grading (++, +, −), which represents the quality as well as the validity of each study included. Studies with all or most checklist criteria fulfilled, indicating overall high quality and a low risk of bias were graded ++, studies with most or some of the checklist criteria fulfilled were graded with a single plus (+), and finally, studies with a high risk of bias and conclusions that are likely to alter were graded with a minus (−). The quality score for each study can be viewed in Supplementary file 2.

## 3. Results

### 3.1. Characteristics of the reviewed studies

Three of the studies had a qualitative research design: Helbekkmo et al. (47), Hirvonen et al. (48), and van Lieshout et al. (49), while 13 of the studies had a quantitative research design (50–62). Three of the quantitative studies were randomized controlled trials: Escribano et al. (50), Pakarinen et al. (51), and Ponsford et al. (52), two applied cluster-randomized controlled designs: de Lijster et al. (53) and Espada et al. (60), and four applied a quasi-experimental design: Elliot et al. (54), García-Vázquez et al. (55), Zmyj and Wehlig (61), and Peters et al. (62). Finally, four studies applied a descriptive design only reporting on post-intervention results: Alekseeva et al. (56), Başar et al. (57), Heras et al. (58), and Pakarinen et al. (59).

The studies included are conducted in different parts of Europe. Four studies were conducted in Southern Europe [Spain (48, 49, 53, 57)], seven in Western Europe [the Netherlands (51, 55, 56); Germany (57); the United Kingdom (47, 51, 54)], three in Northern Europe [Finland (50, 61); Norway (46)], and finally two studies were conducted in Eastern Europe [Turkey (59); Russia (58)]. The age of the participants in the studies ranged from 11 to 19 years [the age range in the study by (56) was 13–23, but age 23 represented the participants’ age at final follow-up]. Most studies were conducted in high schools/secondary schools (47–50, 52–55, 57, 58, 60–62), and only two studies were conducted in vocational schools (51, 59). In one study, the intervention was conducted in both high school and upper secondary schools/vocational schools (56).

### 3.2. Intervention format and content

Alekseeva et al. (56) focused on taboos and stigma as well as the prevention and promotion of sexual health and rights, and a healthy lifestyle. The intervention *Dance4life* was conducted in four different cities in Russia, targeting adolescents between 13 and 19 years old, and encompassed four consecutive stages. No further information on how long the intervention lasted was given. Başar et al. (57), like Alekseeva et al. (56), focused on reproductive health and rights, however, the program also centered around the adolescent period, physiology, reproductive system anatomy, and prevention of STI:s. The study by Başar et al. (57) was conducted in a secondary school in Turkey, with adolescents between 11 and 14 years as the target group. The intervention lasted for a total of 14 h, and separate sessions were arranged for girls and boys during the whole intervention.

Peters et al. (62) reported on an intervention called “Multiple Choice 4 U” in the Netherlands that targeted adolescents in grade 7 (median age 13.5 years). The intervention took place in classrooms during 10 lessons and focused on three psychosocial and behavioral determinants: outcome expectancies (e.g., short-term physical and social consequences), social influences (e.g., social norms), and self-efficacy (e.g., negotiation skills). The *Benzies & Batchies* intervention, studied by de Lijster et al. (53), was also conducted in the Netherlands, but within vocational and secondary schools with adolescents between 12 and 16 as the target group. The focus of the *Benzies & Batchies* intervention was to prevent sexual harassment by focusing on skills and resilience training regarding social and sexual behavior. The intervention consisted of 3×100 min lessons, 30 min play, and 60 min discussion, as well as introductory and closing lessons. In another study (54), the intervention likewise focused on sexual health skills training in an intervention called *Healthy Respect 2 (HR2)*. Along with skills training, the program additionally focused on knowledge and attitudes. The intervention was conducted in 12 high schools in Scotland by specially trained teachers, and the average number of delivered sessions was 22. Adolescents participating in this study were aged 15–16 years old. Hirvonen et al. (48) evaluated the STASH-intervention, which was also set in the United Kingdom. The intervention lasted 10 weeks and focused on online peer support (sharing sexual health messages on Facebook) among adolescents aged 14–16 years old.

Escribano et al. (50) examined the effect of the *COMPAS*-intervention conducted in high schools in Spain among students aged 14–16 years. The focus of the intervention was sexual health promotion as well as HIV prevention. The intervention content covered five components: information, social skills, training, problem-solving skills, and strategies to maintain safer sexual behaviors. Another study, Espada et al. (60), also evaluated the *COMPAS*-intervention in Spain, comparing it to the *¡Cuı ´date*-intervention, which has a similar focus as COMPAS, but with a specific session on communication and negotiation skills. The interventions were conducted in 18 public high schools in Spain. The intensity of the interventions was quite similar, as the COMPAS-intervention consisted of five 50 min sessions and the *¡Cuı ´date*-intervention consisted of six 45 min sessions. Furthermore, García-Vázquez et al. (55) also conducted their intervention study in Spain. The intervention, “Neither Ogres nor Princesses” (NONP), aimed to promote sexual rights and respect for others and to generate conditions for adolescents to make autonomous responsible decisions regarding sexual health and rights. The intervention was delivered by teachers over 4 years with a total of 20.7 h of sex education as well as 7.5 h of workshops.

Helbekkmo et al. (47) evaluated Norwegian students’ experience with the «Week 6» program. The program targeted 15- and 16-year-old adolescents, and the focus of the program was positive sexuality. The program lasted 1 week and focused on different learning goals regarding sexuality according to the school curriculum. Another study, Heras et al. (58), likewise focused on the positive aspects of sexuality and sexual health by highlighting and supporting students’ development of skills needed to maintain a healthy lifestyle and well-being through the SOMOS-intervention. The study was conducted in Spain and consisted of eight sessions that lasted between 12 and 50 min. The target group in the study was 13–19-year-olds.

In the study by Pakarinen et al. (51) the intervention focused on Finnish adolescents’ attitudes, knowledge, and sexual behavior. The intervention targeted adolescents aged 15–19 years and lasted 11 weeks. Another study conducted in Finland by Pakarinen et al. (59) evaluated the same intervention. Thus, the intervention duration and focus were the same as in Pakarinen et al. (51). However, the target group in Pakarinen et al. (59) was first-year students in vocational schools aged 15–16 years old, and the study focused on the participants’ self-evaluation and experiences of the intervention. Moreover, the study by Ponsford et al. (52), like Pakarinen et al. (51), analyzed students’ experiences of and preferences regarding sex education and evaluated the *Positive Choices* program. The focus of the intervention was knowledge, attitudes, and skills. The intervention was conducted in secondary schools in the United Kingdom (participants aged 13–14 years) for one academic year.

Van Lieshout et al. (49) evaluated the Long Live Love + (LLL+) intervention in the Netherlands. The intervention target group was adolescents aged 15–17 years, and the focus was on four different themes: relationships, (un)safe sex and contraception, (un)safe sex and STIs, and sexual diversity, with a duration of two 45-min sessions per theme. Similarly to Van Lieshout et al. (49), the study by Zmyj and Wehling (61) encompassed the topic of sexual diversity, more specifically homonegativity. The workshop was part of the school curriculum in a high school in Germany, and the focus was on changing attitudes toward LGBT-people by encouraging acceptance and respect. The workshop lasted 4 h, and the target group was adolescents aged 14–16 years old.

### 3.3. Focus areas of the interventions

In the narrative synthesis of the studies, five focus areas of the interventions were identified, reflecting both intervention content and related outcomes: (i) attitudes toward sexual health (50, 51, 53, 55, 58, 60, 62), (ii) sexual health awareness (54, 55, 57, 62), (iii) skills related to sexual health (50, 51, 53, 55, 62), (iv) norm perception in relation to sexuality and sexual health (50, 51, 53, 54, 61) and (v) students’ perception of the intervention program (47–49, 52, 56, 59). The attitudes category includes attitudes toward sexual health in general, as well as attitudes toward safe sex, condom use, media influence, and masturbation. The awareness category represents knowledge, awareness, and information on sexual health outcomes. The third category, skills, covers general self-efficacy, self-efficacy in condom use, self-efficacy in communication as well as sexual self-esteem. The fourth category contains outcomes connected to norm perception in relation to sexuality and sexual health, e.g., feelings of social acceptance, acceptability of same-sex relationships, perceived norm, as well as attitudes toward gender roles. The last category highlights students’ perception of the program (e.g., students’ perception of content, implementation of the intervention, and their learning progress). See Table 1 for an overview of the included studies and the categorization of outcomes.

**Table 1:** Key information and focus areas of the programs.

References	Intervention program	Intensity of the intervention	Focus area of the intervention	Statistically significant outcomes related to focus areas	Follow-up
Alekseeva et al. (56)	Dance4life (D4L)	Four stages (no further information)	Perception of the program	Perception of the program +	N
Başar et al. (57)	Reproductive Health Education Program	14 h (2 h per week for 7 weeks)	Awareness	Sexual health knowledge +	Y (2 months)
de Lijster et al. (53)	Benzies and Batchies	3 × 100–150 min + 30 min play, 60 min discussion +2 lessons	Attitudes, skills, norm perception	Sexual self-esteem +	Y (6 months)
Elliot et al. (54)	Healthy Respect 2 (HR2)	Around 22 sessions	Attitudes, awareness, norm perception	Sexual health knowledge +, acceptability of condom use (F) +, intention to use condoms (M) +	Y (24 months)
Escribano et al. (50)	COMPAS	5 × 50-min sessions	Attitudes, skills, norm perception	Attitudes toward condom use +, −when barriers exist +, self-efficacy +	Y (24 months)
Espada et al. (60)	(a) COMPAS and (b) *¡Cuı ´date*	(a) 5 × 50-min (b) 6 × 45 min sessions	Attitudes	No statistically significant effects	Y (24 months)
García-Vázquez et al. (55)	NONP	20–30 h (during a 4-year period)	Attitudes, awareness, skills	Sexual health knowledge +	Y (24 months)
Helbekkmo et al. (47)	«Week 6»	One week	Perception of the program	Perception of the program* +	Y (24 months)
Heras et al. (58)	SOMOS	12 × 50 min sessions	Attitudes	Attitudes toward sexuality +, negative attitudes toward masturbation −	N
Hirvonen et al. (48)	STASH	10 weeks	Perception of the program	Perception of the program +	N
Pakarinen et al. (51)	Sexual health promotion intervention	11 weeks	Attitudes, skills, norm perception	Total attitudes 0, social acceptance of condom use –, self-efficacy in communication –	Y (3 months)
Pakarinen et al. (59)	Sexual health promotion intervention	11 weeks	Perception of the program	Perception of the program +	N
Peters et al. (62)	Multiple Choice 4 U	10 classroom sessions	Attitudes, awareness, skills	No statistically significant effects	Y (4 months)
Ponsford et al. (52)	Positive Choices	One academic year	Perception of the program	Perception of the program* +	Y (12 months)
Van Lieshout, et al. (49)	Long Live Love+ (LLL+)	8 × 45 min sessions	Perception of the program	Perception of the program* +	N
Zmyj and Wehlig (61)	Workshop against homonegativity	4 h	Norm perception	No statistically significant effects	Y (6 weeks)

*qualitative based study.

+ = statistically significant increase; − = statistically significant decrease; 0 = mean scores at baseline and follow-up identical.

F, female; M, male.

### 3.4. Effects of the interventions analyzed utilizing quantitative methods

Ten of the studies with a quantitative design included in this review showed evidence of effect (50–59). The results of the study by Alekseeva et al. (56) showed that 100% of the respondents had improved their knowledge of sexual reproductive health and rights (SRHR) at the post-intervention measurement, 89% reported improved knowledge on how to communicate about sex and sexuality, and 87% had learned to discuss sensitive issues. Moreover, the results showed perceived improvements in skills. The respondents reported greater responsibility for their actions and choices and improvements in communication. Finally, the results indicated an increase in legal literacy related to sexual health among the respondents. The study by Başar et al. (57) showed statistically significant effects on sexual health knowledge for the intervention group at the follow-up, 2 months after the intervention had ended, and there was a statistically significant difference between comparisons between intervention and control group. Furthermore, the results of the study by de Lijster et al. (53) showed a significant improvement in sexual self-esteem for the intervention group.

In the study by Elliot et al. (54), several statistically significant changes were found, showing both positive and negative effects. There was a significant increase in sexual health knowledge for both boys and girls. Group and time interaction was also significant regarding sexual health knowledge, although for girls only. Moreover, there was a significant increase in the intention to use condoms for boys in the intervention group (but not for girls). There was furthermore a statistically significant increase regarding the acceptability to use condoms, although it should be noted that in this specific case, the numerical increase indicates an actual negative development in the acceptability of condom use, showing that girls in the intervention group became less acceptable of condom use. No significant changes were found in the follow-up measurement for boys regarding this outcome, nor was any improvement regarding the acceptability of same-sex relationships found.

In the study by Escribano et al. (50), there were statistically significant positive changes in attitudes toward condom use, attitudes toward condom use when barriers exist, and self-efficacy for the intervention group at the follow-up measurement. Furthermore, the intervention group in the study by García-Vázquez et al. (55) showed significant improvement in sexual health knowledge as well as in skills, but there was only a significant difference at the first post-test measurement regarding the latter outcome. In addition to these studies, Heras et al. (58) showed that there was a statistically significant increase in positive attitudes toward sexual health in general for the intervention group, although with a small effect size, as well as a significant drop in negative attitudes toward masturbation, with a moderate size effect. There was no follow-up measurement, only a post-test measurement in this study. In the study by Pakarinen et al. (51), a statistically significant effect was found for total attitudes, however, the effect concerns differences in mean scores between baseline and post-test and also between post-test and follow-up. The mean baseline and follow-up scores were identical, which means that the results of this study should be interpreted with caution. Regarding the sub-dimensions of total attitudes, no significant effects were found for self-efficacy in condom use. Statistically significant effects were found for social acceptance of condom use and self-efficacy in communication. A small decrease in these outcomes was found from baseline to follow-up. No statistically significant effects or differences were found for the control group.

Pakarinen et al. (59) aimed to examine students’ self-evaluations of a sexual health promotion intervention. Most of the students agreed with the following statements regarding the implementation of the lessons: the content of the lessons was easy to understand (62% agreed), and there was a possibility to ask questions during lessons (53.7% agreed). Finally, Ponsford et al. (52) assessed the feasibility and acceptability of the Positive Choices-intervention, and according to the intervention group, the program covers most of the positive sexual health topics quite well. The topics of sexual consent (IG 82.9%; agreed; CG 62.2% agreed), masturbation (IG 47.4% agreed; CG 15% agreed), love (IG 49.4% agreed; CG 24.7% agreed), sexual pleasure (IG 60.1% agreed; CG 24.7% agreed), readiness for intimacy (IG 41.6% agreed; CG 20.6% agreed), and sexual rights (IG 53.9% agreed; CG 25.8% agreed) are considered to be covered to a higher degree in the intervention group when compared to the control group. Three quantitative studies did not report any statistically significant intervention effects on any of the outcomes of interest in this review (6, 13, 16).

### 3.5. Effects of the interventions analyzed utilizing qualitative methods

The three studies with a qualitative design—Helbekkmo et al. (47), Hirvonen et al. (48), and van Lieshout et al. (49)—all showed good evidence of effect. The overall theme of the qualitative content analysis in the study by Helbekkmo et al. (47) was that the students “liked «Week 6» but had expected more about sex in the sex week.” Subthemes indicate that the students wanted to learn about realistic and relevant subjects as well as to be able to contribute to the content and implementation of the program. Categories that emerged in analyses were (i) organization and content, (ii) positive experiences (iii) potential for improvement, (iv) learning outcome. Hirvonen et al. (48) investigated opportunities and challenges with peer-to-peer sexual health education through social media messages. A total of 35% of the participants were happy to be part of the social media groups and take part of the messages. Among peer supporters, the training helped sensitize them to web-based sexual health content more generally. Messages with humor content, brief, clear texts, memes, and pictures, as well as bold colors, were appreciated by the participants. The participants rarely commented on the posts in general. Some students expressed disinterest in the STASH posts or looked at them out of boredom, and others responded with openness and interest. Finally, pupils in the study by van Lieshout et al. (49) showed more knowledge on contraceptives, and confidence in discussing condoms. Furthermore, participants showed more positive attitudes and confidence in testing and more knowledge of STIs overall. Finally, participants showed more understanding of diversity among LGBT persons and empathy toward persons identifying as LGBT.

## 4. Discussion

This review study offers a rare, if not the first, synthesis of evidence on European school-based sexual health promotion interventions and related effects. Intervention studies applying different designs were included to cover all relevant studies published between 2012 and 2022. Most of the studies included applied quantitative research methods (13/16), while three studies had a qualitative research design providing a more in-depth understanding of the student’s perception of the interventions. While this review aimed to include sexual health promotion interventions, the fact that only 17 publications fulfilled the review inclusion criteria highlights the limited number of school-based sexual health promotion programs in the European context. All included studies had a health promotion aim or content, and all outcomes of interest are connected to the promotion of health resources. However, some studies included have a combined prevention and promotion approach, which should be considered when interpreting the review results. The distinction between the promotion and prevention perspectives is more distinguishable in theory, i.e., practical health promotion work oftentimes encompasses both perspectives (63). Nonetheless, some of the studies included can be acknowledged as representing a universal health promotion approach and therefore serve as good examples for future research and implementation (47, 51, 52, 55, 58, 59).

The review findings provide an overview of sexual health intervention program content evaluated in the European school context. The identified focus areas of the interventions included attitudes toward sexual health, sexual health awareness, skills related to sexual health, norm perception in relation to sexuality and sexual health, and finally, students’ perception of the program. Attitudes toward sexual health (7/16) was the focus area most frequently studied, followed by students’ perception of the program (6/16), skills related to sexual health (5/16), and norm perception in relation to sexuality and sexual health (5/16). Sexual health awareness (4/16) was the least studied focus area. Previous research and theory highlight the importance of a positive approach to sexual health (14, 21–23), and there are, according to Kågesten and Reeuwijk (14) several key competencies that are particularly important for the development of adolescent sexual health and well-being. These competencies are sexual literacy, gender-equitable attitudes, respect for human rights and understanding of consent, critical reflection skills, coping skills, stress management and interpersonal relationship skills. Whereas some of these competencies were prevalent in the studies included in this review (e.g., gender equitable attitudes and interpersonal skills such as communication skills), there are still several competencies lacking in intervention studies (e.g., sexual health literacy, critical reflection skills, and coping- and stress management skills). Sexual health literacy was a key interest in this study since it is a critical part of sexual health promotion in general and has been conceptualized as an important determinant of adolescents’ sexual well-being (14). Regardless of its importance to health promotion, none of the included studies focused on comprehensive, multi-dimensional sexual health literacy *per se*. However, some outcomes of interest found in this review can be seen as a part of the broader health literacy concept, e.g., knowledge, attitudes, self-efficacy, and communication skills (56, 59). Communication is, for instance, an important part of the health literacy concept (13). Therefore, it is possible to draw a parallel to sexual health literacy even though it was not mentioned as a focal point in either of the studies. Based on the lack of focus on the comprehensive sexual health literacy concept in the studies included in this review, it can be recommended to be studied and included in future intervention studies.

A total of 12 out of 16 studies included in this review showed a positive effect in one or more outcomes of interest at the post-test/follow-up measurement or reported positive results based on interviews conducted with the intervention participants. The majority of the quantitative studies included reported at least one statistically significant positive change in outcomes of interest for the intervention group at the post-intervention or follow-up measurements. Several studies (53, 55, 57) reported a statistically significant effect on one outcome of interest. One study (54) reported two statistically significant positive effects, however, a significant change with regard to the acceptability of condom use for girls in the intervention group was also found, showing a negative development of girls’ acceptability of condom use. Therefore, the effect of this intervention is somewhat ambiguous. One study (58) reported two statistically significant positive changes, showing good evidence of effects, while another study (50) reported a total of three positive changes. Hence, considering the studies included in this review the latter study (50), can be regarded as the most promising intervention as it reports beneficial effects for multiple sexual health outcomes. The study was a randomized controlled trial with sexual health promotion intervention content. However, it employed a combined prevention and promotion approach and should, therefore, not be seen as the ideal health promotion intervention. On the other hand, the results of this study were followed-up up 24 months after the intervention had ended, which further strengthens the evidence. The quantitative studies with a descriptive design also indicated positive effects at the post-intervention measurement. One study (59) showed that the students in the intervention group experienced the intervention content and lessons as easy to understand. The results of another study (52) showed that the intervention covered positive sexual health content (e.g., readiness for intimacy, consent, love) very well compared to the control group. Finally, one study (56) showed that the students’ sexual health knowledge, sexual communication skills, and legal literacy skills related to sexual health as well as a responsibility for their actions regarding sexual health, were improved at the post-intervention measurement.

All included studies utilizing qualitative methods (47–49) reported that the participants experienced improvements in sexual health after the intervention, thus, all showed some evidence of effect. However, Helbekkmo et al. (47) also highlighted a gap in content and implementation when compared to what the students had expected. Students’ participation in the planning of sexual health promotion interventions has been proven to be important for engagement and learning (8), which was also highlighted and called for in two of the studies applying interview methods in this review. Helbekkmo et al. (47) reported that students wished to be more involved in planning content and implementing the intervention. Furthermore, Hirvonen et al. (48) reported that the students who were more engaged in the intervention and message sharing seemed to learn and appreciate the intervention more than those who just received the messages. Additionally, and in accordance with previous research (5, 17), this review also highlights students’ wish for a more sex-positive approach in sexual health interventions. One study (47), for instance, concluded that the student’s perception of the “Week 6” program was good but that they had expected more content about sex in a program about positive sexuality.

### 4.1. Strengths and limitations

This review synthesizes findings from quantitative as well as qualitative original studies. Thus, there is a variation in both study and intervention design in the included studies providing a more nuanced and holistic overview of the evidence base. Relevant records were systematically searched for in seven scientific databases. The quality of the records included was evaluated according to the NICE checklist, and the recommendations by JBI were followed. Most of the included studies were deemed to display a low risk of bias, and the conclusions of the review are therefore unlikely to alter (only one study (56), was considered to display a high risk of bias). However, some limitations to this study should be considered when interpreting the results. First, there is a risk that relevant research published in languages other than English was overlooked and excluded. Europe, when compared to, e.g., the USA, is very diverse in languages. Thus, the fact that only articles written in English were included in this review study might also be a possible reason for the lack of diversity in the countries included. Second, gray literature could have been searched in order to find additional articles. Nonetheless, searching for gray literature poses a challenge in relation to systematicity and replicability. Third, there were challenges related to determining the health intervention approach (i.e., risk approach or health-resource approach) for the studies in the study selection process. Studies that were considered to solely apply a risk perspective were excluded in order to align with the review aim. However, after several critical discussions, studies encompassing positive sexual health outcomes but applying a mixed prevention and promotion intervention approach were included in the data synthesized. Another important limitation to consider when interpreting the results is that the data this review is based on did not allow for a statistical meta-analysis of the intervention effects, which would have provided more robust statistical calculations on the pooled effectiveness in addition to the narrative evidence synthesis.

### 4.2. Conclusion

The evidence synthesis provides an overview of holistic and resource-focused sexual health interventions conducted in European schools, reflecting an emerging sexual health promotion approach that goes beyond the prevention of STIs and unplanned pregnancies – and instead highlights the importance of equality, respect, communication skills, attitudes toward sexual health and other positive and strengthening sexual health resources. The majority of the included studies showed evidence of statistically significant effects on at least one of the health promotion outcomes of interest, and a few studies even showed positive effects on multiple outcomes and can therefore be considered good examples of sexual health promotion interventions. The study also contributes with an overview of the intervention and outcome focus areas in the sexual health promotion interventions in Europe. The existing programs cover attitudes toward sexual health and students’ perception of the program well. However, there is a lack of attentive focus on the comprehensive, multi-dimensional sexual health literacy concept, despite its importance for positive sexual health development for adolescents. Thus it can be recommended to be studied further and included in future intervention studies.

## Author contributions

RA formulated the aim of the study and the applied inclusion and exclusion criteria, conducted searches in the selected databases, screened the records found, and conducted the data extraction, as well as quality appraisal and data synthesis and also wrote the initial manuscript draft, revised it, and edited it. JN participated in the formulations of aim and eligibility criteria, the article screening process, and the discussions around study selection and coding of the retrieved data. JN furthermore conducted the quality appraisal together with RA and reviewed and revised all the manuscript draft versions. AF participated in the formulations of aim and eligibility criteria as well as in the discussions regarding the final selection of included studies and related coding and also critically reviewed the various versions of the manuscript. All authors contributed to the article and approved the submitted version.

## Funding

This study was financed by Åbo Akademi University Foundation, which had no impact on the content of this manuscript.

## Conflict of interest

The authors declare that the research was conducted in the absence of any commercial or financial relationships that could be construed as a potential conflict of interest.

## Publisher’s note

All claims expressed in this article are solely those of the authors and do not necessarily represent those of their affiliated organizations, or those of the publisher, the editors and the reviewers. Any product that may be evaluated in this article, or claim that may be made by its manufacturer, is not guaranteed or endorsed by the publisher.
