# Supplementary material for: Promoting sexual health in schools: a systematic review of the European evidence
Source: Front Public Health. 2023 Jul 4;11:1193422. doi: 10.3389/fpubh.2023.1193422 (PMC10352496; doi:10.3389/fpubh.2023.1193422)
Supplement: Supplementary file 1 [file Table_1.DOCX]

Supplementary Material

Promoting Sexual Health in Schools: A Systematic Review of the European Evidence

Ronja Abrams*, Johanna Nordmyr, Anna K. Forsman

*** Correspondence:** Corresponding Author: [ronja.abrams@abo.fi](mailto:ronja.abrams@abo.fi)

**Supplementary File 1: Search Strategies**

**PubMed**

| Searches | Search terms (AB/TI, MeSH 2012-2022) | Results |
| --- | --- | --- |
| #1 | ("Sexual health"[MeSH Terms] OR "sexuality"[MeSH Terms] OR "Sex education"[MeSH Terms] OR "sexual health/education"[MeSH Terms] OR "sexual behavior"[MeSH Terms] OR "Sexual health"[Title/Abstract] OR "sexual health promot*"[Title/Abstract] OR "sexual well*"[Title/Abstract] OR "holistic sexual health"[Title/Abstract] OR "sexual litera*"[Title/Abstract] OR "sexuality"[Title/Abstract] OR "Sex education"[Title/Abstract] OR "sexuality education"[Title/Abstract] OR "sexual health/education"[Title/Abstract] OR "comprehensive sexuality education"[Title/Abstract]) | **55 092** |
| #2 | ("adolescent"[MeSH Terms] OR "students"[MeSH Terms] OR "schools"[MeSH Terms] OR "student*"[Title/Abstract] OR "adolescent*"[Title/Abstract] OR "young adult*"[Title/Abstract] OR "teen*"[Title/Abstract] OR "young people"[Title/Abstract] OR "youth"[Title/Abstract] OR "girls"[Title/Abstract] OR "boys"[Title/Abstract] OR "young women"[Title/Abstract] OR "young men"[Title/Abstract] OR "school*"[Title/Abstract]) | **1 048 673** |
| #3 | ("program evaluation"[MeSH Terms] OR "intervention*"[Title/Abstract] OR "initiative*"[Title/Abstract] OR "program*"[Title/Abstract] OR "action*"[Title/Abstract] OR "evaluat*"[Title/Abstract] OR "implement*"[Title/Abstract] OR "enhanc*"[Title/Abstract] OR "promot*"[Title/Abstract]) | **4 530 045** |
| #4 | "European Union" OR Europe* OR "EU-27" OR "European country" OR "European countries" OR Austria OR Belgium OR Bulgaria OR Cyprus OR "Czech Republic" OR Denmark OR Estonia OR Finland OR France OR Germany OR Greece OR Hungary OR Ireland OR Italy OR Latvia OR Lithuania OR Luxembourg OR Malta OR Netherlands OR Holland OR Poland OR Portugal OR Romania OR Slovak* OR Slovenia OR Spain OR Sweden OR "United Kingdom" OR England OR Wales OR Scotland OR "Great Britain" OR Croatia OR "Former Yugoslav Republic of Macedonia" OR Macedonia OR Iceland OR Montenegro OR Turkey OR Albania OR Andorra OR Armenia OR Azerbaijan OR Belarus OR "Bosnia and Herzegovina" OR Bosnia OR Georgia OR Liechtenstein OR Moldova OR Monaco OR Norway OR Russia OR "San Marino" OR Serbia OR Switzerland OR Ukraine OR (Vatican AND (City OR State)) OR European* OR Austrian* OR Belgian* OR Bulgarian* OR Cypriot* OR Czech* OR Danish* OR Estonian* OR Finnish* OR French* OR German* OR Greek* OR Hungarian* OR Irish* OR Italian* OR Latvian* OR Lithuanian* OR Luxembourg* OR Maltese* OR Dutch* OR Hollander* OR Netherlander* OR Polish* OR Portuguese* OR Romanian* OR Slovak* OR Slovenian* OR Spanish* OR Swedish* OR English* OR Scottish* OR Britannic* OR British* OR Welsh* OR Croatian* OR Macedonian* OR Icelandic* OR Turkish* OR Albanese* OR Andorran* OR Armenian* OR Azerbaijani* OR Belarusian* OR Bosnian* OR Georgian* OR Liechtenstein OR Moldavian* OR Monaco OR Nordic* OR Norwegian* OR Russian* OR Serbian* OR Swiss* OR Ukrainian* OR Vatican* | **810 280** |
| #5 | #1 AND #2 AND #3 | **11 660** |
| #6 | #1 AND #2 AND #3 AND #4 | **1 533** |

*** *Searches conducted and downloaded to Zotero 01.04.2022*

**Web of Science**

| Searches | Search terms (AB, TI, TS 2012-2022) | Results |
| --- | --- | --- |
| #1 | ((TI=(“Sexual health” OR “sexual health promot*” OR “sexual well*” OR “holistic sexual health” OR “sexual litera*” OR sexuality OR “Sex education” OR “sexuality education” OR “sexual health education” OR “comprehensive sexuality education” )) OR TS=(“Sexual health” OR “sexual health promot*” OR “sexual well*” OR “holistic sexual health” OR “sexual litera*” OR sexuality OR “Sex education” OR “sexuality education” OR “sexual health education” OR “comprehensive sexuality education” )) OR AB=(“Sexual health” OR “sexual health promot*” OR “sexual well*” OR “holistic sexual health” OR “sexual litera*” OR sexuality OR “Sex education” OR “sexuality education” OR “sexual health education” OR “comprehensive sexuality education” ) | **38 286** |
| #2 | ((TS=(Student* OR adolescent* OR “young adult*” OR teen* OR “young people” OR youth OR girls OR boys OR ”young women” OR ”young men” OR School*)) OR TI=(Student* OR adolescent* OR “young adult*” OR teen* OR “young people” OR youth OR girls OR boys OR ”young women” OR ”young men” OR School*)) OR AB=(Student* OR adolescent* OR “young adult*” OR teen* OR “young people” OR youth OR girls OR boys OR ”young women” OR ”young men” OR School*) | **1 305 528** |
| #3 | ((TS=(Intervention* OR initiative* OR program* OR action* OR evaluat* OR implement* OR enhanc* OR promot*)) OR TI=(Intervention* OR initiative* OR program* OR action* OR evaluat* OR implement* OR enhanc* OR promot*)) OR AB=(Intervention* OR initiative* OR program* OR action* OR evaluat* OR implement* OR enhanc* OR promot*) | **9 051 578** |
| #4 | AB OR TI OR TS: ("European Union" OR Europe* OR "EU-27" OR "European country" OR "European countries" OR Austria OR Belgium OR Bulgaria OR Cyprus OR "Czech Republic" OR Denmark OR Estonia OR Finland OR France OR Germany OR Greece OR Hungary OR Ireland OR Italy OR Latvia OR Lithuania OR Luxembourg OR Malta OR Netherlands OR Holland OR Poland OR Portugal OR Romania OR Slovak* OR Slovenia OR Spain OR Sweden OR "United Kingdom" OR England OR Wales OR Scotland OR "Great Britain" OR Croatia OR "Former Yugoslav Republic of Macedonia" OR Macedonia OR Iceland OR Montenegro OR Turkey OR Albania OR Andorra OR Armenia OR Azerbaijan OR Belarus OR "Bosnia and Herzegovina" OR Bosnia OR Georgia OR Liechtenstein OR Moldova OR Monaco OR Norway OR Russia OR "San Marino" OR Serbia OR Switzerland OR Ukraine OR (Vatican AND (City OR State)) OR European* OR Austrian* OR Belgian* OR Bulgarian* OR Cypriot* OR Czech* OR Danish* OR Estonian* OR Finnish* OR French* OR German* OR Greek* OR Hungarian* OR Irish* OR Italian* OR Latvian* OR Lithuanian* OR Luxembourg* OR Maltese* OR Dutch* OR Hollander* OR Netherlander* OR Polish* OR Portuguese* OR Romanian* OR Slovak* OR Slovenian* OR Spanish* OR Swedish* OR English* OR Scottish* OR Britannic* OR British* OR Welsh* OR Croatian* OR Macedonian* OR Icelandic* OR Turkish* OR Albanese* OR Andorran* OR Armenian* OR Azerbaijani* OR Belarusian* OR Bosnian* OR Georgian* OR Liechtenstein OR Moldavian* OR Monaco OR Nordic* OR Norwegian* OR Russian* OR Serbian* OR Swiss* OR Ukrainian* OR Vatican*) | **2 421 690** |
| #5 | #1 AND #2 AND #3 | **5 814** |
| #6 | #1 AND #2 AND #3 AND #4 | **966** |

*** *Searches conducted and downloaded to Zotero 01.04.2022*

**SCOPUS**

| Searches | Search terms (AB/TI/KEYWORD 2012-2022) | Results |
| --- | --- | --- |
| #1 | **“**Sexual health” OR “sexual health promot*” OR “sexual well*” OR “holistic sexual health” OR “sexual litera*” OR sexuality OR **“**Sex education” OR “sexuality education” OR “sexual health education” OR “comprehensive sexuality education” | **59 909** |
| #2 | Student* OR adolescent* OR “young adult*” OR teen* OR “young people” OR youth OR girls OR boys OR ”young women” OR ”young men” OR school* | **2 478 248** |
| #3 | Intervention* OR initiative* OR program* OR action* OR evaluat* OR implement* OR enhanc* OR promot* | **11 098 174** |
| #4 | "European Union" OR Europe* OR "EU-27" OR "European country" OR "European countries" OR Austria OR Belgium OR Bulgaria OR Cyprus OR "Czech Republic" OR Denmark OR Estonia OR Finland OR France OR Germany OR Greece OR Hungary OR Ireland OR Italy OR Latvia OR Lithuania OR Luxembourg OR Malta OR Netherlands OR Holland OR Poland OR Portugal OR Romania OR Slovak* OR Slovenia OR Spain OR Sweden OR "United Kingdom" OR England OR Wales OR Scotland OR "Great Britain" OR Croatia OR "Former Yugoslav Republic of Macedonia" OR Macedonia OR Iceland OR Montenegro OR Turkey OR Albania OR Andorra OR Armenia OR Azerbaijan OR Belarus OR "Bosnia and Herzegovina" OR Bosnia OR Georgia OR Liechtenstein OR Moldova OR Monaco OR Norway OR Russia OR "San Marino" OR Serbia OR Switzerland OR Ukraine OR (Vatican AND (City OR State)) OR European* OR Austrian* OR Belgian* OR Bulgarian* OR Cypriot* OR Czech* OR Danish* OR Estonian* OR Finnish* OR French* OR German* OR Greek* OR Hungarian* OR Irish* OR Italian* OR Latvian* OR Lithuanian* OR Luxembourg* OR Maltese* OR Dutch* OR Hollander* OR Netherlander* OR Polish* OR Portuguese* OR Romanian* OR Slovak* OR Slovenian* OR Spanish* OR Swedish* OR English* OR Scottish* OR Britannic* OR British* OR Welsh* OR Croatian* OR Macedonian* OR Icelandic* OR Turkish* OR Albanese* OR Andorran* OR Armenian* OR Azerbaijani* OR Belarusian* OR Bosnian* OR Georgian* OR Liechtenstein OR Moldavian* OR Monaco OR Nordic* OR Norwegian* OR Russian* OR Serbian* OR Swiss* OR Ukrainian* OR Vatican* | **7 965 639** |
| #5 | #1 AND #2 AND #3 | **11 830** |
| #6 | #1 AND #2 AND #3 AND #4 | **3 997** |

** Searches conducted and downloaded to Zotero 01.04.2022*

**CINAHL**

| Searches | Search terms (AB, TI, KW, MH 2012-2022) | Results |
| --- | --- | --- |
| #1 | ( (MH "Sexual Health") OR "sexual health" OR (MH "Sex Education") OR "sex education" OR (MH "Sexuality") OR "sexuality" ) OR TI ( “Sexual health” OR “sexual health promot*” OR “sexual well*” OR “holistic sexual health” OR “sexual litera*” OR sexuality OR “Sex education” OR “sexuality education” OR “sexual health education” OR “comprehensive sexuality education” ) OR AB ( “Sexual health” OR “sexual health promot*” OR “sexual well*” OR “holistic sexual health” OR “sexual litera*” OR sexuality OR “Sex education” OR “sexuality education” OR “sexual health education” OR “comprehensive sexuality education” ) | **32 177** |
| #2 | ( (MH "Schools") OR "schools" OR (MH "Adolescence") OR "adolescents" OR (MH "Students") OR "students" ) OR TI ( Student* OR adolescent* OR “young adult*” OR teen* OR “young people” OR youth OR girls OR boys OR ”young women” OR ”young men” OR “school class” OR school* ) OR AB ( Student* OR adolescent* OR “young adult*” OR teen* OR “young people” OR youth OR girls OR boys OR ”young women” OR ”young men” OR school* ) | **569 959** |
| #3 | ( "intervention" OR (MH "Experimental Studies") OR (MH "Program Evaluation") OR (MH "Program Implementation") OR "program" ) OR TI ( Intervention* OR initiative* OR program* OR action* OR evaluat* OR implement* OR enhance* OR promot* ) OR AB ( Intervention* OR initiative* OR program* OR action* OR evaluat* OR implement* OR enhanc* OR promot* ) | **1 318 757** |
| #4 | AB OR TI: "European Union" OR Europe* OR "EU-27" OR "European country" OR "European countries" OR Austria OR Belgium OR Bulgaria OR Cyprus OR "Czech Republic" OR Denmark OR Estonia OR Finland OR France OR Germany OR Greece OR Hungary OR Ireland OR Italy OR Latvia OR Lithuania OR Luxembourg OR Malta OR Netherlands OR Holland OR Poland OR Portugal OR Romania OR Slovak* OR Slovenia OR Spain OR Sweden OR "United Kingdom" OR England OR Wales OR Scotland OR "Great Britain" OR Croatia OR "Former Yugoslav Republic of Macedonia" OR Macedonia OR Iceland OR Montenegro OR Turkey OR Albania OR Andorra OR Armenia OR Azerbaijan OR Belarus OR "Bosnia and Herzegovina" OR Bosnia OR Georgia OR Liechtenstein OR Moldova OR Monaco OR Norway OR Russia OR "San Marino" OR Serbia OR Switzerland OR Ukraine OR (Vatican AND (City OR State)) OR European* OR Austrian* OR Belgian* OR Bulgarian* OR Cypriot* OR Czech* OR Danish* OR Estonian* OR Finnish* OR French* OR German* OR Greek* OR Hungarian* OR Irish* OR Italian* OR Latvian* OR Lithuanian* OR Luxembourg* OR Maltese* OR Dutch* OR Hollander* OR Netherlander* OR Polish* OR Portuguese* OR Romanian* OR Slovak* OR Slovenian* OR Spanish* OR Swedish* OR English* OR Scottish* OR Britannic* OR British* OR Welsh* OR Croatian* OR Macedonian* OR Icelandic* OR Turkish* OR Albanese* OR Andorran* OR Armenian* OR Azerbaijani* OR Belarusian* OR Bosnian* OR Georgian* OR Liechtenstein OR Moldavian* OR Monaco OR Nordic* OR Norwegian* OR Russian* OR Serbian* OR Swiss* OR Ukrainian* OR Vatican* | **345 706** |
| #5 | #1 AND #2 AND #3 | **6 372** |
| #6 | #1 AND #2 AND #3 AND #4 | **737** |

** Searches made through EBSCO-host*

** Searches conducted and downloaded to Zotero 01.04.2022*

**ERIC**

| Searches | Search terms (AB, TI, DE 2012-2022) | Results |
| --- | --- | --- |
| #1 | ( (DE "Sex Education") OR (DE "Sexuality") ) OR TI ( “Sexual health” OR “sexual health promot*” OR “sexual well*” OR “holistic sexual health” OR “sexual litera*” OR sexuality OR “Sex education” OR “sexuality education” OR “sexual health education” OR “comprehensive sexuality education” ) OR AB ( “Sexual health” OR “sexual health promot*” OR “sexual well*” OR “holistic sexual health” OR “sexual litera*” OR sexuality OR “Sex education” OR “sexuality education” OR “sexual health education” OR “comprehensive sexuality education” ) | **3 114** |
| #2 | ( ((DE "Students") OR (DE "Schools")) OR (DE "Adolescents") ) OR TI ( Student* OR adolescent* OR “young adult*” OR teen* OR “young people” OR youth OR girls OR boys OR ”young women” OR ”young men” OR school* ) OR AB ( Student* OR adolescent* OR “young adult*” OR teen* OR “young people” OR youth OR girls OR boys OR ”young women” OR ”young men” OR school*) | **286 758** |
| #3 | ( ((((DE "Intervention") OR (DE "Program Effectiveness")) OR (DE "Program Evaluation")) OR (DE "Program Implementation")) OR (DE "Action Research") ) OR TI ( Intervention* OR initiative* OR program* OR action* OR evaluat* OR implement* OR enhanc* OR promot* ) OR AB ( Intervention* OR initiative* OR program* OR action* OR evaluat* OR implement* OR enhanc* OR promot* ) | **216 755** |
| #4 | TI OR AB: ("European Union" OR Europe* OR "EU-27" OR "European country" OR "European countries" OR Austria OR Belgium OR Bulgaria OR Cyprus OR "Czech Republic" OR Denmark OR Estonia OR Finland OR France OR Germany OR Greece OR Hungary OR Ireland OR Italy OR Latvia OR Lithuania OR Luxembourg OR Malta OR Netherlands OR Holland OR Poland OR Portugal OR Romania OR Slovak* OR Slovenia OR Spain OR Sweden OR "United Kingdom" OR England OR Wales OR Scotland OR "Great Britain" OR Croatia OR "Former Yugoslav Republic of Macedonia" OR Macedonia OR Iceland OR Montenegro OR Turkey OR Albania OR Andorra OR Armenia OR Azerbaijan OR Belarus OR "Bosnia and Herzegovina" OR Bosnia OR Georgia OR Liechtenstein OR Moldova OR Monaco OR Norway OR Russia OR "San Marino" OR Serbia OR Switzerland OR Ukraine OR (Vatican AND (City OR State)) OR European* OR Austrian* OR Belgian* OR Bulgarian* OR Cypriot* OR Czech* OR Danish* OR Estonian* OR Finnish* OR French* OR German* OR Greek* OR Hungarian* OR Irish* OR Italian* OR Latvian* OR Lithuanian* OR Luxembourg* OR Maltese* OR Dutch* OR Hollander* OR Netherlander* OR Polish* OR Portuguese* OR Romanian* OR Slovak* OR Slovenian* OR Spanish* OR Swedish* OR English* OR Scottish* OR Britannic* OR British* OR Welsh* OR Croatian* OR Macedonian* OR Icelandic* OR Turkish* OR Albanese* OR Andorran* OR Armenian* OR Azerbaijani* OR Belarusian* OR Bosnian* OR Georgian* OR Liechtenstein OR Moldavian* OR Monaco OR Nordic* OR Norwegian* OR Russian* OR Serbian* OR Swiss* OR Ukrainian* OR Vatican*) | **81 114** |
| #5 | #1 AND #2 AND #3 | **1 254** |
| #6 | #1 AND #2 AND #3 AND #4 | **144** |

** Searches made through EBSCO-host*

*** *Searches conducted and downloaded to Zotero 01.04.2022*

**Education Research Complete**

| Searches | Search terms (AB, TI, DE, KW 2012-2022) | Results |
| --- | --- | --- |
| #1 | DE "SEX education" OR TI ( “Sexual health” OR “sexual health promot*” OR “sexual well*” OR “holistic sexual health” OR “sexual litera*” OR sexuality OR “Sex education” OR “sexuality education” OR “sexual health education” OR “comprehensive sexuality education” ) OR AB ( “Sexual health” OR “sexual health promot*” OR “sexual well*” OR “holistic sexual health” OR “sexual litera*” OR sexuality OR “Sex education” OR “sexuality education” OR “sexual health education” OR “comprehensive sexuality education” ) | **5 311** |
| #2 | ((DE "STUDENTS") OR (DE "SCHOOLS")) OR (DE "TEENAGERS") ) OR TI ( Student* OR adolescent* OR “young adult*” OR teen* OR “OR “young people” OR youth OR girls OR boys OR ”young women” OR ”young men” OR ”High school seniors” OR school* ) OR AB ( Student* OR adolescent* OR “young adult*” OR teen* OR “OR “young people” OR youth OR girls OR boys OR ”young women” OR ”young men” OR school* ) | **480 697** |
| #3 | ( DE "EDUCATIONAL intervention" OR DE "PROGRAM implementation (Education)" OR DE "PROGRAM development (Education)" OR DE "PROGRAM improvement (Education)" OR DE "PROGRAM effectiveness (Education)" ) OR TI ( Intervention* OR initiative* OR program* OR action* OR evaluat* OR implement* OR enhanc* OR promot* ) OR AB ( Intervention* OR initiative* OR program* OR action* OR evaluat* OR implement* OR enhanc* OR promot* ) | **362 374** |
| #4 | AB OR TI: "European Union" OR Europe* OR "EU-27" OR "European country" OR "European countries" OR Austria OR Belgium OR Bulgaria OR Cyprus OR "Czech Republic" OR Denmark OR Estonia OR Finland OR France OR Germany OR Greece OR Hungary OR Ireland OR Italy OR Latvia OR Lithuania OR Luxembourg OR Malta OR Netherlands OR Holland OR Poland OR Portugal OR Romania OR Slovak* OR Slovenia OR Spain OR Sweden OR "United Kingdom" OR England OR Wales OR Scotland OR "Great Britain" OR Croatia OR "Former Yugoslav Republic of Macedonia" OR Macedonia OR Iceland OR Montenegro OR Turkey OR Albania OR Andorra OR Armenia OR Azerbaijan OR Belarus OR "Bosnia and Herzegovina" OR Bosnia OR Georgia OR Liechtenstein OR Moldova OR Monaco OR Norway OR Russia OR "San Marino" OR Serbia OR Switzerland OR Ukraine OR (Vatican AND (City OR State)) OR European* OR Austrian* OR Belgian* OR Bulgarian* OR Cypriot* OR Czech* OR Danish* OR Estonian* OR Finnish* OR French* OR German* OR Greek* OR Hungarian* OR Irish* OR Italian* OR Latvian* OR Lithuanian* OR Luxembourg* OR Maltese* OR Dutch* OR Hollander* OR Netherlander* OR Polish* OR Portuguese* OR Romanian* OR Slovak* OR Slovenian* OR Spanish* OR Swedish* OR English* OR Scottish* OR Britannic* OR British* OR Welsh* OR Croatian* OR Macedonian* OR Icelandic* OR Turkish* OR Albanese* OR Andorran* OR Armenian* OR Azerbaijani* OR Belarusian* OR Bosnian* OR Georgian* OR Liechtenstein OR Moldavian* OR Monaco OR Nordic* OR Norwegian* OR Russian* OR Serbian* OR Swiss* OR Ukrainian* OR Vatican* | **179 097** |
| #5 | #1 AND #2 AND #3 | **1 364** |
| #6 | #1 AND #2 AND #3 AND #4 | **177** |

** Searches made through EBSCO-host*

*** *Searches conducted and downloaded to Zotero 01.04.2022*

**PsycINFO**

| Searches | Search terms (AB, TI, DE, 2012-2022) | Results |
| --- | --- | --- |
| #1 | ( ((DE "Sexual Health") OR (DE "Sexuality")) OR (DE "Sex Education") ) OR TI ( “Sexual health” OR “sexual health promot*” OR “sexual well*” OR “holistic sexual health” OR “sexual litera*” OR sexuality OR “Sex education” OR “sexuality education” OR “sexual health education” OR “comprehensive sexuality education” ) OR AB ( “Sexual health” OR “sexual health promot*” OR “sexual well*” OR “holistic sexual health” OR “sexual litera*” OR sexuality OR “Sex education” OR “sexuality education” OR “sexual health education” OR “comprehensive sexuality education” ) | **19 443** |
| #2 | ( ((DE "Students") OR (DE "Adolescent Health")) OR (DE "Schools") ) OR TI ( Student* OR adolescent* OR “young adult*” OR teen* OR “young people” OR youth OR girls OR boys OR ”young women” OR ”young men” OR school* ) OR AB ( Student* OR adolescent* OR “young adult*” OR teen* OR “young people” OR youth OR girls OR boys OR ”young women” OR ”young men” OR school* ) | **443 554** |
| #3 | ( ((((DE "Intervention") OR (DE "School Based Intervention")) OR (DE "Program Development")) OR (DE "Program Evaluation")) OR (DE "Action Research") ) OR TI ( Intervention* OR initiative* OR program* OR action* OR evaluat* OR implement* OR enhanc* OR promot* ) OR AB ( Intervention* OR initiative* OR program* OR action* OR evaluat* OR implement* OR enhanc* OR promot* ) | **832 241** |
| #4 | AB OR TI: "European Union" OR Europe* OR "EU-27" OR "European country" OR "European countries" OR Austria OR Belgium OR Bulgaria OR Cyprus OR "Czech Republic" OR Denmark OR Estonia OR Finland OR France OR Germany OR Greece OR Hungary OR Ireland OR Italy OR Latvia OR Lithuania OR Luxembourg OR Malta OR Netherlands OR Holland OR Poland OR Portugal OR Romania OR Slovak* OR Slovenia OR Spain OR Sweden OR "United Kingdom" OR England OR Wales OR Scotland OR "Great Britain" OR Croatia OR "Former Yugoslav Republic of Macedonia" OR Macedonia OR Iceland OR Montenegro OR Turkey OR Albania OR Andorra OR Armenia OR Azerbaijan OR Belarus OR "Bosnia and Herzegovina" OR Bosnia OR Georgia OR Liechtenstein OR Moldova OR Monaco OR Norway OR Russia OR "San Marino" OR Serbia OR Switzerland OR Ukraine OR (Vatican AND (City OR State)) OR European* OR Austrian* OR Belgian* OR Bulgarian* OR Cypriot* OR Czech* OR Danish* OR Estonian* OR Finnish* OR French* OR German* OR Greek* OR Hungarian* OR Irish* OR Italian* OR Latvian* OR Lithuanian* OR Luxembourg* OR Maltese* OR Dutch* OR Hollander* OR Netherlander* OR Polish* OR Portuguese* OR Romanian* OR Slovak* OR Slovenian* OR Spanish* OR Swedish* OR English* OR Scottish* OR Britannic* OR British* OR Welsh* OR Croatian* OR Macedonian* OR Icelandic* OR Turkish* OR Albanese* OR Andorran* OR Armenian* OR Azerbaijani* OR Belarusian* OR Bosnian* OR Georgian* OR Liechtenstein OR Moldavian* OR Monaco OR Nordic* OR Norwegian* OR Russian* OR Serbian* OR Swiss* OR Ukrainian* OR Vatican* | **1 927 004** |
| #5 | #1 AND #2 AND #3 | **3 327** |
| #6 | #1 AND #2 AND #3 AND #4 | **3 340** |

** Searches made through EBSCO-host*

*** *Searches conducted and downloaded to Zotero 01.04.2022*
