# Supplementary material for: Promoting sexual health in schools: a systematic review of the European evidence
Source: Front Public Health. 2023 Jul 4;11:1193422. doi: 10.3389/fpubh.2023.1193422 (PMC10352496; doi:10.3389/fpubh.2023.1193422)
Supplement: Supplementary file 2 [file Table_2.DOCX]

Supplementary Material

Promoting Sexual Health in Schools: A Systematic Review of the European Evidence

Ronja Abrams*, Johanna Nordmyr and Anna K. Forsman

*** Correspondence:** Corresponding Author: [ronja.abrams@abo.fi](mailto:ronja.abrams@abo.fi)

**Supplementary File 2: Overview of Key Information from Studies Included in the Systematic Review**

| **1. Alekseeva, et al., (2014)** | | | | | | |
| --- | --- | --- | --- | --- | --- | --- |
| **First author and year:**  Alekseeva, E., (2014)  **Country of study:**  Russia  **Aim of study:**  To study the effectiveness of the international volunteer program dance4life (D4L)  **Study design:**  Descriptive  **Quality assessment score:**  - | **Setting:**  4 Russian cities  **Participants:**  In the quantitative research (N = 105): 48 % were aged 13-16, 44 % were aged 17-19, and 8 % were aged 20-23. Both boys and girls were included.  Six focus group interviews and in-depth interviews with 13-19-year-olds. Snowball method recruitment  **Inclusion:**  Participants who completed three or four stages of the program.  Sample generated via random selection from various schools  **Exclusion** (reasons listed):  Not reported | **Intervention(s):**  Dance4life (D4L) aims to address taboos, stigma, discrimination, HIV/AIDS prevention, and the promotion of sexual reproductive health and rights (SRHR) and a healthy lifestyle among adolescents. The program uses an “edutainment” model involving young people through music, dance, and icons. The program encompasses four consecutive stages.  **Control:**  No control groups  **Sample sizes:**  Qualitative data: n = 36  Six focus groups (six participants per group): two groups with girls, two with boys, and two with mixed groups; 20 in-depth interviews.  Quantitative data: n = 105 | | **Outcome measures of interest:** *Questionnaire content:*  (1) personal effects of the program and personal openness and discussion of sensitive issues with significant others  (2) awareness of sexual and reproductive rights  (3) knowledge and discrimination practices - perception and experience; stigma attitude aspiration.  **Interview guide:**  *Interview questions:* program impact on changes in personal life, knowledge, and skills; assessment of their organization of volunteer activities in the program; sharing knowledge and experience from the program with others.  **Follow-up periods:**  Only post-intervention data was collected.  **Method of analysis:**  Qualitative data: no form of analysis was reported. Quantitative data: only frequencies (%) reported | **Key results:**  **Awareness***:* 100 % knew more about SRHR, 87 % knew how to discuss sensitive issues such as HIV/AIDS, and 89 % could talk more openly about sexuality.  **Skills and behavior:** They had a growing responsibility for their actions and choices. They were also able to develop their communication and organizational skills. 48 % have informed others about rights, and 87 % have learned to talk about sex and sexuality.  **Stigma and taboo:** young people indicated increased legal literacy, feeling that the program had helped them know about their legal and other SRH rights and the mechanisms to implement and protect them. 96% reported they would not stigmatize people living with HIV. | **Limitations (author):**  (1) The sample is only those who completed all stages of the program; 20 % of the overall population (2) Limited sample size (3) Not possible to conclude the long-term impact of the program (4) No pre-post measurement comparison |
| **2. Başar, et al., (2021)** | | | | | | |
| **First author and year:**  Başar, F. (2021)  **Country of study:**  Turkey  **Aim of study:**  This study aimed to determine the effectiveness of a reproductive health education program for adolescents.  **Study design:**  Quasi-experimental  **Quality assessment score:**  **+** | **Setting:**  A secondary school in Kütahya, Turkey  **Participants:**  161 adolescents aged 11 to 14 years.  **Inclusion:**  Adolescents between 11 and 14 years old, with reading- and writing literacy.  The classes were selected by a simple random sampling method (coin toss) and assigned to the control or intervention condition.  **Exclusion** (reasons listed):  Not reported | **Intervention(s):**  A reproductive health education program for 14 hours (2 hours per week for 7 weeks). Separate sessions for boys and girls. Content: (1) Introduction, meeting the students (2) Adolescent period, changes in the adolescent period (3) reproductive system anatomy and physiology (4) Risky behavior in the adolescent period (5) Sexually transmitted diseases and prevention (6) Reproductive health, reproductive rights (7) Closing, general evaluation. In this program, the researcher educated girls and boys in separate groups.  **Control:**  The control group received reproductive health education after they completed the post-test  **Sample sizes:**  IG: n = 84  CG: n = 77  Power analysis was performed, a sample size of 128 was deemed sufficient based on a type I error rate of 5%, a medium effect size of 0.25, and a power ratio of 80%. | | **Outcome measures of interest:**  The reproductive health information form consisted of 33 questions measuring:  (1) the level of knowledge regarding changes in the adolescent period  (2) reproductive health and reproductive rights of adolescents  Correct answers awarded 1 point (total of 33 points possible, range 0-33)  **Follow-up periods:**  Post-measurement at six weeks after training for both IG and CG.  **Method of analysis:**  T-test was used to evaluate the students' knowledge levels in the intervention and control groups before and after the education. The level of statistical significance was accepted as α=0.05. | **Key results:**  **Reproductive health score averages:**  Pre-test scores, mean (SD):  IG: 17.97 (5.22)  CG: 18.18 (5.28)  Between-group comparison  t = 0.248, p = 0.804  Post-test scores, mean (SD):  IG: 27.51 (3.83)  CG: 18.36 (5.88)  Between-group comparison  t = -11.579, *p* < 0.001  Pre-test – post-test comparison  IG: t = -18.537, *p* < 0.001  CG: t = -0.338, *p* = 0.737 | **Limitations (author):**  (1) randomization could not be done by sample selection. (2) The study was conducted at a single center in Turkey. (3) Due to the short duration of the educational program (seven weeks), the long-term impact of the program and its sustainability could not be assessed. (4) IG and CG differed in economic status. |
| **3. de Lijster, et al., (2016)** | | | | | | |
| **First author and year:**  de Lijster, G., (2016)  **Country of study:**  The Netherlands  **Aim of study:**  To evaluate the effectiveness of Benzies & Batchies, an interactive school-based program to prevent male and female adolescent sexual harassment behavior in secondary school students by combining play with skills lessons and peer education.  **Study design:**  Cluster-randomized controlled trial  **Quality assessment score:**  ++ | **Setting:**  28 pre-vocational and senior general secondary education in urban areas in the Netherlands. The schools were part of the mainstream regular Dutch school system.  **Participants:**  Adolescents aged 12–16 years from various ethnic backgrounds  Schools were paired according to educational level and the degree of urbanization of the school area. The schools were then randomly assigned to the experimental or control condition.  **Inclusion:**  Not reported  **Exclusion** (reasons listed):  Not reported | **Intervention(s):**  Benzies & Batchies consisted of four complementary elements: (a) an introductory lesson, (b) an educational peer-performed play followed by a peer-led group discussion, (c) three classroom lessons, each 100–150 min, to teach skills and resilience regarding social and sexual behavior; and (d) a closing lesson.  **Control:**  Waiting list CG  **Sample sizes:**  Baseline, 747 respondents:  IG: n = 403  CG: n = 344  Post-test, 694 respondents (93 %):  IG: n = 397  CG: n = 297  Follow-up, 621 respondents (83 %):  IG: n = 326  CG: n = 295 | | **Outcome measures of interest:**  (1) Attitude towards gender roles: 12-item scale (Hofstetter et al. 2014).  (2) Attitude towards media influence: 8-item scale (De Graaf et al. 2009; Nikken 2007).  (3) Sexual self-esteem*:* 7-item scale (Rostosky et al. 2008)  **Follow-up periods:** Post-test and a 6-month follow-up  **Method of analysis:**  Multilevel analyses were conducted to obtain the effects of the intervention at the first post-test and 6-month follow-up. A two-level random intercept model was used, with students at first level and school at second level. | **Key results:**  *** indicates p <0.01*  **Attitude towards gender roles**  IG:  Baseline: 45.07 (7.56)  Post-test: 45.93 (8.02)  Follow-up: 46.16 (9.19)  Difference score (T2–T0) = 0 .97 (8.36)  CG:  Baseline: 43.58 (8.66)  Follow-up: 45.41 (9.33)  Difference score (T2–T0) = 2.05 (9.55)  β=-0.13  Effect size = -0.12  **Attitude toward media influence**  IG:  Baseline: 28.46 (5.82)  Post-test: 29.53 (6.23)  Follow-up: 29.56 (6.80)  Difference score (T2–T0) = 0.92 (6.46)  CG:  Baseline: 27.30 (6.61)  Post-test: 27.82 (6.42)  Follow-up: 27.94 (7.04)  Difference score (T2–T0) = 0.89 (7.21)  β= 0.09  Effect size = 0.00  **Sexual self-esteem**  IG:  Baseline: 30.90 (5.31)  Post-test: 31.50 (5.46)  Follow-up: 31.81 (4.99)  Difference score (T2–T0) = 1.36 (5.97)  CG:  Baseline: 30.57 (5.02)  Post-test: 30.19 (5.62)  Follow-up: 30.34 (6.03)  Difference score (T2–T0) = -0.43 (6.46)  β= 0.34**  Effect size = 0.29 | **Limitations (author)**  (1) As not all students reported having sexual experiences, they may not have been able to imagine being in a situation of sexual harassment and/or being interested in having a relationship or sex. (2) Some questionnaire scales were developed or adapted for the purpose of this part. (3) Students’ self- reports were used on a delicate subject; the prevalence rates may have been underreported: students might have found it difficult to report having committed sexual harassment or having been a victim of it. (4) This study was conducted in schools in an urban setting. Different results may be produced by research into the effectiveness of the intervention in schools in non-urban areas. |
| **4. Elliot, et al., (2012)** | | | | | | |
| **First author and year:**  Elliot, L. (2012)  **Country of study:**  United Kingdom  **Aim of study:**  To examine whether the Healthy Respect 2 HR2 program improved young people’s sexual health knowledge, attitudes, behavior and use of sexual health services and reduced socioeconomic inequalities in sexual health.  **Study design:**  Quasi-experimental  **Quality assessment score:**  + | **Setting:**  12 high schools in Scotland (6 in the intervention area, 6 in the control area)  **Participants:**  6608 pupils aged 15– 16 years, 5283 pupils included in the data analysis  **Inclusion:**  Students aged 15-16 years old  **Exclusion** (reasons listed):  (1) Missing sexual health outcome data or spoiled questionnaires, and (2) those absent from school because they were attending college and vocational courses, sick, truanting, suspended, permanently excluded, or attending alternate education settings (15%). | **Intervention(s):**  Specially trained teachers in the HR2 program delivered a theoretically based sex education program (SHARE). The average number of sessions delivered to pupils was 22, of which 33% included skills training.  **Control:**  Sexual health education in CG schools consisted mainly of information and discussion (average number of sessions 8, no skills training offered). Teachers were not specially trained.  **Sample sizes:**  IG: 2269 (1111 females and 1158 males)  CG: 3014 (1442 females and 1572 males)  Sample size (when missing data etc. excluded):  Baseline:  IG: n= 814  CG: n= 1030  Second survey:  IG: n = 760  CG: n = 987  Third survey  IG: n= 695  CG: n= 997  The study power estimated at 80% power to detect an increase from 81% to 91% for females, and from 69% to 79% for males in the outcome “intentions to use condoms.” | | **Outcome measures of interest:**  (1) A total knowledge score compromised the sum of correct responses to 11 questions on practical sexual health information. (2) Tolerance of same-sex relationships was based on an agreement to three statements using a five-point scale where 1= ‘most acceptable’.  **Follow-up periods:**  2007 (baseline), 2008 and 2009 (combined post-intervention surveys).  **Method of analysis:**  Regression modelling was used to assess the differences between the intervention and comparison groups at baseline, post-intervention, and over time. Due to the serial cross-sectional design, difference-in-differences (dif-in-difs) analyses were conducted using logistic and linear regression for dichotomous and continuous outcomes. | **Key results:**  n.s = not significant  **Sexual health knowledge:**  Girls:  Pre-test scores: mean, β, (SE):  IG: 7.08, β=0.00,  CG: 7.25, β=0.00  Combined post-intervention scores: mean, β, (SE):  IG: 7.39, β=0.35 (0.14), p<0.05  CG: 6.92, β=0.30 (0.11), p<0.01  Group-time interaction β, (SE):  β=0.56 (0.18), p<0.01  Boys:  Pre-test scores: mean, β, (SE):  IG: 6.90, β=0.00  CG: 6.78, β= 0.00  Combined post-intervention scores: mean, β, (SE):  IG: 7.18, β=0.43 (0.13), p<0.001  CG: 6.58, β= –0.29 (0.12), p<0.05  Group-time interaction β, (SE):  β=0.72 (0.18), p<0.001  **Acceptability of same-sex relationships:**  Girls:  Pre-test scores: mean, β, (SE):  IG: 2.86, β =0.00  CG: 2.94, β=0.00  Combined post-intervention scores: mean, β, (SE):  IG: 2.82, β=−0.04 (0.05), n.s.  CG: 3.01, β= 0.11 (0.05), p<0.05  Group-time interaction  β (SE)=-0.15 (0.07), p<0.05  Boys:  Pre-test scores: mean, β, (SE):  IG: 3.29, β=0.00  CG: 3.48, β=0.00  Combined post-intervention scores: mean, β, (SE):  IG: 3.34, β=0.04 (0.06), n.s.  CG: 3.51, β=0.05 (0.06), n.s.  Group-time interaction  β (SE)=0.00 (0.09), n.s.  **Acceptability of condom use**  Girls:  Pre-test scores: mean, β, (SE):  IG: 1.92, β =0.00  CG: 1.99, β=0.00  Combined post-intervention scores: mean, β, (SE):  IG: 2.04, β=−0.15 (0.04), p<0.01  CG: 2.04, β= 0.05 (0.04) n.s.  Group-time interaction  β (SE)= 0.11 (0.06), p<0.01  Boys:  Pre-test scores: mean, β, (SE):  IG: 2.16, β=0.00  CG: 2.15, β=0.00  Combined post-intervention scores: mean, β, (SE):  IG: 2.13, β=0.01 (0.04) n.s.  CG: 2.12, β=–0.01 (0.04) n.s.  Group-time interaction  β (SE)=0.01 (0.06), n.s.  **Intends to use condoms**  Girls:  Pre-test scores: mean, β, (SE):  IG: 87.5, β =0.00  CG: 86.0, β=0.00  Combined post-intervention scores: mean, β, (SE):  IG: 85.2, β=−0.99 (0.87–1.13), n.s.  CG: 83.1, β= 0.85 (0.60–1.19) n.s.  Group-time interaction  β (SE)=-1.09 (0.64 to 1.86) n.s.  Boys:  Pre-test scores: mean, β, (SE):  IG: 81.7, β=0.00  CG: 81.8, β=0.00  Combined post-intervention scores: mean, β, (SE):  IG: 82.5, β=1.00 (0.69 to 1.45), n.s.  CG: 76.5, β=0.69 (0.51 to 0.95), p<0.05  Group-time interaction  β (SE)= 1.44 (0.89 to 2.32), n.s.  Analyses were also conducted controlling for the effect of socioeconomic status. | **Limitations (author):**  (1) The use of dif-in-difs in the analysis leads to potential overestimation of SEs. However, a clustered analysis design shrinks the SEs, and thus, mitigates against the likelihood that significant effects were found when they did not exist. (2) those at greater sexual risk may have been missed from the intervention arm. (3). The more rigorous the evaluation design, the less likely it is to find positive outcomes. (4) The intervention components designed for vulnerable young people were less developed compared with those for mainstream educational settings. |
| **5. Escribano, et al., (2015)** | | | | | | |
| **First author and year:**  Escribano, et.al., (2015)  **Country of study:**  Spain  **Aim of study:**  To determine the factors that mediate in the self-reported consistent condom use over the 24-months post-intervention period in adolescents who received COMPAS, a sexual health promotion intervention.  **Study design:**  Randomized control trial  **Quality assessment score:**  **+** | **Setting:**  Twelve high schools were located in the north, east, and south of Spain.  **Participants:**  1121 adolescents aged 14–16 (50.7 % girls; average age = 14.76, SD = 0.75). The participants were 9th and 10th grade high school students at the beginning of the study. They participated in the trial over a 25-month period.  **Inclusion:**  Not reported  **Exclusion** (reasons listed):  Not reported | **Intervention(s):**  COMPAS is a school intervention promoting sexual health and HIV prevention based on the Social Learning Theory and the Information-Motivation-Behavioral Skills model (IMB). Main program components: information, social skills training, problem-solving training, and strategies to maintain safer sexual behavior (5 x 50-min sessions).  **Control:**  No intervention  **Sample sizes:**  IG: 622  CG: 499  Six schools were randomly assigned to the COMPAS group (n = 622). The remaining six schools formed the control group. | | **Outcome measures of interest:**  (1) Attitude toward condom use was evaluated with the 3-item  Attitude toward condom use subscale from the 12-item  HIV Attitudes Scale for Adolescents  (2) Self-efficacy was evaluated with the General Self-Efficacy Scale with Spanish adolescents. It is comprised  of 10 items with 10-point Likert response scale  (3) Perceived norms: was evaluated with the item, ‘‘How frequently do you believe that your peers use condoms in their sexual relations,’’ with a Likert-type response scale of 1–4.  **Follow-up periods:**  Pre- and post-intervention assessment, 12 and 24-month follow-up assessments.  **Method of analysis:**  Serial multiple mediator models using the procedures described by Hayes. The predictor was a dummy variable contrasting the sexual health promotion intervention (COMPAS) with the control group. | **Key results:**  Effect of intervention on variables of interest reported here  **Attitude toward condom use**  Pre-test scores: mean (SE):  IG: 13.09 (2.31)  CG: 13.05 (2.56)  Post-intervention scores: mean (SE):  IG: 14.02 (2.27)  CG: 14 (2.27)  Effect: 0.65 (0.23), 95% CI 0.19, 1.10, p=0.005  **Attitude toward condom use when barriers exist**  Pre-test scores: mean (SE):  IG: 9.50 (2.16)  CG: 9.30 (2.23)  Post-intervention scores: mean (SE):  IG: 9.78 (2.29)  CG: 9.94 (2.13)  Effect: 0.49 (0.20), 95% CI 0.09, 0.90, p=0.015  **(2) Self-efficacy**  Pre-test scores: mean (SE):  IG: 69.57 (15.48)  CG: 68.78 (16.46)  Post-intervention scores: mean (SE):  IG: 74.78 (11.97)  CG: 72.49 (11.84)  Effect: 4.06 (1.42), 95% CI 1.26, 6.85, p=0.004  **Perceived norms**  Pre-test scores: mean (SE):  IG: 2.04 (0.83)  CG: 2.03 (0.81)  Post-intervention scores: mean (SE):  IG: 1.98 (0.73)  CG: 1.98 (0.73)  Effect: -0.13 (0.08), 95% CI -0.28, 0.02, p=0.109 | **Limitations (author):**  (1) The intervention’s effect was assessed with a self-report. (2) The participating schools were not recruited by representative sampling, so generalizations must be made with caution. (3) Mediation analyses imply correlation; experimental studies with manipulation of the variables under study would be necessary to be able to conclude causality |
| **6. Espada, et al., (2017)** | | | | | | |
| **First author and year:**  Espada, J., (2017)  **Country of study:**  Spain  **Aim of study:**  To evaluate the effects of the Competencias para adolescentes con una sexualidad saludable (COMPAS) program and compare them with an evidence-based program (¡Cuıdate!) and a control group (CG)  **Study design:**  Cluster-randomized controlled trial.  **Quality assessment score:**  + | **Setting:**  18 public high schools in north, south, and east Spain.  **Participants:**  1,563 students aged 14–16 in 9th and 10th grades.  **Inclusion:**  Not reported  **Exclusion** (reasons listed):  Not reported | **Intervention(s):**  *COMPAS* is a school-based sexual health education intervention developed and tested in Spain. It consists of five 50min sessions: two sessions are dedicated to addressing knowledge about identifying sexual risk, HIV/AIDS and other STIs, methods of protection, and mistaken beliefs. The third session addresses e.g., decisions related to condom use. The last two modules are about communication skills and the ability to negotiate in several sex situations.  *¡Cuı ́date:* Its curriculum consists of six 45-min sessions. The first three sessions are about HIV/AIDS and concepts associated with its prevention. The fourth and fifth sessions address condom use (barriers, skills for correct use). The last sessions are about communication skills.  **Control:**  No intervention  **Sample sizes:**  *COMPAS* intervention group: 6 schools *(n* = 622). *¡Cuídate!* Intervention group: 6 schools *(n* = 442)  Control group: 6 schools (*n* = 499) | | **Outcome measures of interest:**  (1) Intention to engage in safe sex: This evaluated the intention to engage in safer sexual behavior over 12 months with 5 items.  **Follow-up periods:**  24 months after the implementation  **Method of analysis:**  Generalized estimating equations (GEE)  To test the 24-month follow-up effects of COMPAS, one contrast compared COMPAS with the CG, and another contrast compared COMPAS with ¡Cuı ́date! To test the 24-month follow-up effects of ¡Cuı ́date!, a contrast compared ¡Cuı ́date! with the CG. | **Key results:**  **Intention to engage in safe sex**:  Baseline:  COMPAS: 4.22 (0.29)  iCU ́IDATE!: 4.19 (0.29)  CG: 4.20 (0.28)  24-months follow up:  COMPAS: 4.18 (0.29)  iCU ́IDATE!: 4.29 (0.29)  CG: 4.22 (0.29)  Effect size:  Intention to engage in safe:  COMPAS—CG:  AORa 1.02 [0.93, 1.13], p=0.567  ¡CU´IDATE! CG  AORb 0.99 [0.89, 1.10], p=0.877  COMPAS—¡CU´IDATE!  AORc 0.96 [0.87, 1.06], p=0.46 | **Limitations (author):**  (1) The high rate of lost participants at 24 months post-intervention. (2) The sole use of a self-report assessment method. |
| **7. García-Vázquez, et al., (2019)** | | | | | | |
| **First author and year:**  Garcia-Vázquez, J.G. (2019)  **Country of study:**  Spain  **Aim of study:**  To examine whether the program “Neither Ogres Nor Princesses” (NONP) improved adolescents’ sexuality knowledge, attitudes and skills, and increase condom use.  **Study design:**  Quasi-experimental  **Quality assessment score:**  **+** | **Setting:**  Five secondary schools (out of 21 total with the NONP program)  **Participants:**  656 first-year pupils (12- to 13-year- old)  **Inclusion:**  Sampling was done by cluster, and intervention schools were selected randomly until the established sample size was reached. Comparability was sought in the same context: for each intervention school, another school in the same geographical area that did not participate in NONP or any other sex education program was randomly selected.  **Exclusion** (reasons listed):  (1) Surveys poorly answered, which were less than 1% in each arm and at each time, were excluded. (2) Pupils who had not previously completed the four grades of secondary school in the same center were excluded | | **Intervention(s):**  Teachers in the NONP program taught 20.7h of sex education to their pupils during the 4 years of intervention workshops made up another 7.5h. Pupils received an average of 28.1h (range: 24–34) of intervention. Based on a rights and gender approach the program aims to generate conditions for people to make autonomous responsible decisions and promotes that they can exercise their rights, fulfill their responsibilities, and respect the rights of others.  **Control:**  In control schools, activities during the 4 years accounted for 4.2h on average.  **Sample sizes:**  IG: 327  CG: 329  Post-test 1  IG: 310  CG: 298  Post-test 2  IG: 186  CG: 185  Sample size was determined for 80% statistical power. | **Outcome measures of interest:**  15 items for:  (1) knowledge, (with a choice of a true/ false)  (2) attitudes, (using a Likert-type scale)  (3) skills (ability to do), using frequency scale or ‘yes/no’    **Follow-up periods:**  Post-intervention survey at the end of the intervention (pupils 15- and 16-year-olds); and follow-up 24 months after the intervention (17- and 18-year-olds).  **Method of analysis:**  Difference-in-differences (dif-in-difs) analyses conducted to evaluate intervention impact | **Key results:**  **Knowledge:**  Pre-test scores, mean (SD):  IG  All: 4.1 (0.12)  Girls: 3.9 (0.18)  Boys: 4.1 (0.17)  CG  All: 4.8 (0.12)  Girls: 4.8 (0.18)  Boys: 4.7 (0.18)  Post-test scores, mean (SD):  IG  All: 7.7 (0.14)  Girls: 8.1 (0.18)  Boys: 7.4 (0.22)  CG  All: 7.8 (0.14), p< 0.01  Girls: 7.9 (0.19)  Boys: 7.8 (0.20)  *Dif-in-difs:* 0.32 (0.04, 0.60), p<0.05  **Attitudes:**  Pre-test scores, mean (SD):  IG  All: 5.9 (0.13)  Girls: 6.1 (0.18)  Boys: 5.7 (0.18)  CG  All: 6.2 (0.13)  Girls: 6.4 (0.17)  Boys: 6.1 (0.19)  Post-test scores, mean (SD):  IG  All: 8.3 (0.15)  Girls: 9.1 (0.13)  Boys: 7.5 (0.26)  CG  All: 8.5 (0.14), p< 0.01  Girls: 9.1 (0.15)  Boys: 7.9 (0.22)  *Dif-in-difs:* 0.09 (–.18, 0.37),  p< 0.509  **Skills:**  Pre-test scores, mean (SD):  IG  All: 5.8 (0.12)  Girls: 6.1 (0.17)  Boys: 5.4 (0.16)  CG  All: 6.1 (0.13)  Girls: 6.3 (0.16)  Boys: 5.9 (0.20)  Post-test scores, mean (SD):  IG  All: 7.1 (0.16)  Girls:  Boys:  CG  All: 7.1 (0.15), p< 0.01  Girls: 7.5 (0.19)  Boys: 6.7 (0.23)  *Dif-in-difs:* 0.13 (–.14, 0.41), p<0.349 | **Limitations (author):**  (1) Response rates for pre-test and post-tests were 96%, 92%, and 81%. (2) The quality of the intervention relies on teachers and their experience, training, and fidelity, which were not controlled. (3) One possible bias could be the pupils’ socioeconomic, cultural and religious status (not controlled for) (4) The sample size in the second post-test was smaller than expected, decreasing statistical power. |
| **8. Helbekkmo et al., (2021)** | | | | | | |
| **First author and year:**  Helbekkmo, E. (2021)  **Country of study:**  Norway  **Aim of study:**  To explore adolescents’ experiences with participation in a sexual health education program named «Week 6», from a health-promotion perspective.  **Study design:**  Descriptive  **Quality assessment score**    ++ | **Setting:**  Two different municipalities in the southeast area of Norway  **Participants:**  31 students: 11 boys and 20 girls aged 15–16 from four different classes (10^th^ grade)  **Inclusion:**  To be included, the school must have arranged «Week 6» multiple times. Furthermore, the students must have participated in «Week 6» and attended the same school throughout secondary school.  **Exclusion** (reasons listed):  Not listed | | **Intervention(s):**  In Norway, the program «Week 6» is produced and owned by the organization Sex and Politics and is designed to meet different learning goals from the school’s curriculum about sexuality. «Week 6» consists of the primary program and an additional program with a new theme every year. In 2019, the theme of the additional material was *positive sexuality* (this was not used by the included schools)  **Control:**  No control groups  **Sample sizes:**  6 focus groups with 4–7 participants (who had received the program once or twice) in each group | **Interview guide:**  Semi-structured interviews (open questions focusing on program structure, process, and results)  **Follow-up periods:**  The interviews were conducted 8–10 months after the students’ participation in «Week 6».  **Method of analysis:**  Qualitative content analysis, following the approach of Graneheim and Lundman (2004) | **Key results:**  **Theme:** We like «Week 6», but … “we expected more about sex in the sex week”.  **Subthemes:** (i)The students want a topical sexual health education with realistic and relevant learning subjects and exercises. (ii) The students want to contribute to the content and implementation to improve the learning outcomes of «Week 6»**.**  **Categories:**  (i) Organization and content  (ii) Positive experiences  (iii) Potential for improvement  (iv) Learning outcome  **Subcategories:** e.g., who teaches, classroom environment etc. | **Limitations (author):**  One weakness of the study material is that relies on the informants’ recollections (interviews conducted 8-10 months after the program). |
| **9. Heras, et al., (2016)** | | | | | | |
| **First author and year:**  Heras, D., (2016)  **Country of study:**  Spain  **Aim of study:**  To study the effects of the SOMOS Sexual Education Program on attitudes toward sexuality in general and toward masturbation in particular, as well as the possible impact of that program on the sexual experiences of pupils  **Study design:**  Quasi-experimental  **Quality assessment score:**  + | **Setting:**  Institute of Secondary Education in Castilla y León  **Participants:**  123 students enrolled in the third year: 55 males (44.72%) and 68 females (55.28%), age range 13-17 years (*M* = 14.18, *SD* = .72). Non-probabilistic incidental sample.  **Inclusion:**  Attendance at the educational center  **Exclusion** (reasons listed):  Not reported | | **Intervention(s):**  SOMOS, a Sexual Education program, aims to support students’ development of the skills needed to maintain a healthy lifestyle and wellbeing. The program consists of eight Didactic Units, each developed in 12 50-minute sessions: Unit.1 We are sexual beings!; Unit.2 Our body is changing; Unit.3 We have sexual feelings; Unit.4 We have relation- ships; Unit.5 Our response; Unit.6 We express ourselves; Unit.7 What are the risks?; Unit.8 For our health!  **Control:**  Two control groups (adolescents who had not attended the educational intervention).  **Sample sizes:**  ATSS:  IG: n = 71  CG: n = 52  NA-MI:  IG: n = 67  CG: n = 48 | **Outcome measures of interest:**  (1) Negative Attitudes toward Masturbation Inventory (NA-MI): Abramson and Mosher (1975); a 5-point Likert-type scale formed of 30 items, which evaluate the presence of negative attitudes toward masturbation.  (2) The Attitudes Towards Sexuality Scale (ATSS): Fisher and Hall (1988) enlarged and validated by Diéguez, López, López, & Sueiro (2001). This is a 5-point Likert-type scale that has 28 items  **Follow-up periods:**  Post-test measurement  **Method of analysis:**  Parametric and non-parametric statistics. For the analysis of liberalism in attitudes toward sexuality and negative attitudes towards masturbation, the *T* test for related samples was applied. | **Key results:**  ***** Indicates *p* <0.05  ** Indicates *p* <0.01  **ATSS Attitudes towards Sexuality:**  Pre-test scores, mean (SD):  IG :104.10 (12.11)  CG: 104.64 (14.06)  Post-test scores, mean (SD):  IG: 106.84 (11.98)  t = -2.54, p< 0.013*, d = 0.23  CG: 05.69 (4.814),  t = -0.69, p< 0.492, d = -0.07  **NA-MI Negative attitudes towards Masturbation:**  Pre-test scores, mean (SD):  IG: 75.93 (11.39)  CG: 77.33 (15.11)  Post-test scores, mean (SD):  IG: 71.55 (14.080),  t = 2.76, p<0.007**, d = 0.34  CG: 74.66 (17.900),  t = 1.65, p<0.106, d = 0.17 | **Limitations (author):**  The intervention was limited to twelve sessions and implemented at only one educational center. |
| **10b. Hirvonen, et al. (2021)** | | | | | | |
| **First author and year:**  Hirvonen, M. (2021)  Parallel study: Forsyth et.al. (2018)  **Country of study:**  United Kingdom  **Aim of study:**  To investigate opportunities and challenges of using social media to facilitate peer-to-peer sharing of sexual health messages within the context of STASH (Sexually Transmitted Infections and Sexual Health), a secondary school-based and peer-led sexual health intervention.  **Study design:**  Descriptive  **Quality assessment score:**  + | **Setting:**  Six state-funded secondary schools in Lothian region, Scotland.  **Participants:**  fourth year students aged 14-16 years (42 interview participants, 680 + 88 questionnaire participants)  **Inclusion:**  All S4 students (aged 14–16) at state-funded schools who have received or are currently in receipt of teacher-led sex education, regardless of their sexual experience or individual level of risk are eligible for inclusion.  **Exclusion** (reasons listed):  Private schools | **Intervention(s):**  The STASH (Sexually Transmitted infections and Sexual Health) intervention was adapted from the effective peer-led antismoking ASSIST (A Stop Smoking in Schools Trial) intervention, premised on the diffusion of innovation theory. The intervention recruited, trained, and supported students nominated by their friends to serve as peer supporters. During a 10-week intervention, peer supporters were asked to share sexual health messages from the STASH website among their friends via Facebook (a private group function) and face-to-face conversations. Peer supporters also distributed the URL and password to access the STASH website (printed on cards) to their friends**.**  **Control:**  No control group  **Sample sizes:**  11 group interviews with 42 students (3 female groups, 3 male groups, 5 mixed gender groups)  680 + 88 questionnaire participants | | **Interview guide:**  The interviews probed participants’ experiences and views of the STASH intervention.  **Follow-up periods:**  Interviews conducted during final 2 intervention weeks  No follow-up periods.  Web-based questionnaire completed by student peer supporters at final follow-up session  Web-based questionnaire completed by students at 6-month follow-up  **Method of analysis:**  Thematic analysis of qualitative data using NVivo software. One author coded the entire data set for the process evaluation and one author, using a separate coding frame, coded portions of the data set pertaining to the social media component for the study. Key themes were identified via synthesis of coding and discussion between analysts and with reference to the existing literature.  Descriptive statistics | **Key results:**  **Engagement With Facebook:** Friends who did join found the groups acceptable; in the follow-up questionnaire, 60.2% (91/151) of students who were invited to STASH Facebook groups said they were happy to be a member and 35.0% (55/157) said they learned about sexual health by being part of the group.  **The Offline STASH Context Legitimated and Augmented Web-based Posts**  Among peer supporters, the training also helped sensitize them to web-based sexual health content more generally.  **Preferences for Message Design and Content:** (1) Participants commonly appreciated humor. They felt it helped draw attention and get messages across. (2) Brief, clear text and memes or pictures were viewed as “more interesting than reading like a chart or something like that*...*” (friend, male). Finally, balanced arguments were viewed positively. (3) Bold colors were also viewed as attention grabbing  **Recipients of Messages Are Sometimes Receptive but Do Not Necessarily Engage Visibly on social media**  (1) Some friends expressed disinterest in the STASH posts or looked at them out of boredom, others responded with openness and interest. (2) Even when they were receptive to messages, friends rarely commented substantively on posts. | **Limitations (author):**  (1) The necessity of conducting interviews within school periods-imposed time limits that were shorter than ideal and limited the depth of discussion on social media. (2) Field notes attested to a broader discomfort with discussion of sexual matters. This may have prevented students from admitting to a deeper interest in, or engagement with, the STASH messages. (3) The paired and group interview format may also have influenced individuals’ inclination to discuss their views and experiences, particularly if they differed from others in the group. |
| **11. Pakarinen et al., (2020)** | | | | | | |
| **First author and year:**  Pakarinen, M. (2019)  **Country of study:**  Finland  **Aim of study:**  To describe adolescents’ attitudes, knowledge, and sexual behavior before and after a sexual health promotion intervention.  **Study design:**  Randomized controlled trial  **Quality assessment score:**  **+** | **Setting:**  The study was carried out in eight randomly selected vocational schools in Finland.  **Participants:**  First-year students between 15 and 19 years of age (n = 228). All Finnish vocational schools (n = 228) were divided into four different size groups according to the number of first-year students. One school from each of the four groups was randomly selected as an intervention group and one as a control group.  **Inclusion:**  Not listed  **Exclusion** (reasons listed):  Not listed | | **Intervention(s):**  The intervention was developed in the study and consisted of three elements: (i) a 45-minute class-room session held by the students’ teacher, (ii) five types of information materials, and (iii) free condom distribution in the schools. The duration of the intervention was 11 weeks  **Control:**  Regular curriculum  **Sample sizes:**  Baseline:  IG: n = 500  CG: n = 183  Second follow-up:  IG: n = 202  CG: n = 46 | **Outcome measures of interest:**  Attitudes were measured with 12 statements which were answered using a five-stage Likert scale varying from 1 (fully agree) to 5 (fully disagree).  (1) self-efficacy in condom use  (2) self-efficacy in communication (3) feeling of social acceptance about condom use.  **Follow-up periods:**  The first follow-up measurements were taken immediately after the intervention had ended. The second follow-up measurements were taken 3 months after the intervention had ended.  **Method of analysis:**  Analysis: cross-tabulation and v2 and Kruskal–Wallis H tests. The statistical significance was  set as p < 0.05 | **Key results:**  α= Cronbach’s alpha  **Total attitudes:**  IG: p<0,001  CG: p<0,341  Pre-test scores, mean (SD), α:  IG: 3.9 (0,61), α= 0.752  CG: 3.9 (0,62), α= 0.772,  Second follow-up, mean (SD), α:  IG: 3.9 (0,68), α= 0.804  CG: 3.8 (0,63) α= 0.716  **Self-efficacy in communication:**  IG: p<0.003  CG: p< 0.224  Pre-test scores, mean (SD), α:  IG: 4.1 (0.80) α= 0.551  CG: 4.0 (0.81), α= 0.534  Second follow-up, mean (SD), α:  IG: 4.0 (0.89), α= 0.649  CG: 4.1 0.85 α= 0.536  **Self-efficacy in condom use:**  IG: p< 0.274  CG: p< 0.085  Pre-test scores, mean (SD), α:  IG: 3.8 (0,90) α= 0.591  CG: 3.9 (0,91) α=0.690  Second follow-up, mean (SD), α:  IG: 3.8 (0,92) α= 0.588  CG: 4.1 (0,93) α= 0.659  **Feeling of social acceptance about condom use:**  IG: p<0,003  CG: p<0,086  Pre-test scores, mean (SD), α:  IG: 3.9 (0,86), α= 0.609  CG: 3.9 (0,87), α= 0.677  Second follow-up, mean (SD), α:  IG: 37 (0,97), α= 0.702  CG: 38 (1,05), α=0.764 | **Limitations (author):**  (1) The study was conducted at a group level and individual answers could not be included in the analysis. It is likely that a part of the participants answered only one or two questionnaires. (2) The data included mainly male participants and therefore cannot be applied to all vocational school students in the same age group. (3) There was a lack of participants compared with the baseline in the control follow-up groups, and in the intervention follow-up groups. (4) The first follow-up measurements were taken immediately after the intervention had ended (intervention first follow-up n = 173/control first follow-up n = 115). The second follow-up measurements were taken 3 months after the intervention had ended. |
| **12. Pakarinen et al., (2019)** | | | | | | |
| **First author and year:**  Pakarinen, M. (2019)  **Country of study:**  Finland  **Aim of study:**  To examine students’ self-evaluations of a sexual health promotion intervention carried out in four randomly selected vocational schools.  **Study design:**  Descriptive  **Quality assessment score:**  **+** | **Setting:**  The study was carried out in four (n = 4) randomly selected vocational institutions in Finland.  **Participants:**  First-year students from four (n = 4) randomly selected vocational institutions who had participated in the RCT study described above (Pakarinen et al, 2019a).  **Inclusion:**  Not reported  **Exclusion** (reasons listed):  Not reported | | **Intervention(s):**  The intervention was developed in the study and consisted of three elements as described in Pakarinen et al. 2019a. The duration of the intervention was 11 weeks.  **Control:**  No Control group  **Sample sizes:**  n=168 recruited  Participated in the intervention:  (n = 123, 73.2%) | **Outcome measures of interest:**  Self-evaluation of the classroom lesson “Learning from the lesson“. Five statements were presented concerning learning “a lot of new information about”:  (1) the topics of sexuality,  (2) sex,  (3) Safer sex  A self-completed electronic questionnaire was developed for the study. The evaluation was made with a Likert scale that varied from 1 as fully agree to 5 fully disagree.  **Follow-up periods:**  The self-evaluation was conducted within two weeks after the intervention had ended.  **Method of analysis:**  Connections between variables were examined with cross-tabulations and Pearson’s chi-squared test, and with Kruskal–Wallis H-test, Mann– Whitney U-test, or with Spearman’s rho. The statistical significance was set as p < 0.05 | **Key results:**  α = Chronbach’s alpha  **Self-evaluation of the classroom lesson,** *mean (SD),* α:  Learning: 3.4 (1.2) α= 0.957  I have learned a lot of new information about sexuality:  29,5 % agree, 32,8 % undecided, 37,7 % disagree  I have learned a lot of new information about sex:  26,8% agree, 27,6 % undecided, 45,6 % disagree  I have learned a lot of new information on safe sex:  20,5 % agree, 32 % undecided, 47,5% disagree  **Implementation of the lesson,** mean (SD), α:  Implementation*:* 2.6 (1) α= 0.758  Content of the lesson was easy to understand:  62.6 % agree, 22.8 % undecided, 14,6 % disagree  There was a possibility to ask questions during the lesson:  53.7 % agree, 31.4 % undecided, 14,9 % disagree  Content of the lesson was interesting:  32.8 % agree, 38.5 % undecided, 28,7 % disagree  The mean score of learning from the classroom lesson variable was 3.4 (SD 1.2) | **Limitations (author):**  (1) The participants were mainly male (the data are not representative of all the vocational school students) (2) The response rate cannot be defined because there is no information available on how many students obtained the questionnaire. (3) The data were collected via a self-reported questionnaire administered in a school setting, so there is a potential for misreporting, inaccurate recollection, or other reporting errors. |
| **13. Peters, et al., (2013)** | | | | | | |
| **First author and year:**  Peters, L., (2013)  **Country of study:**  The Netherlands  **Aim of study:**  To evaluate an innovative curriculum about smoking and safe sex that also focused on promoting students’ transfer of knowledge, skills, and attitudes to other domains.  **Study design:**  Quasi-experimental  **Quality assessment score:**  + | **Setting:**  23 secondary schools from all regions of the Netherlands  **Participants:**  The participating students were in Grade 7 (16%) or 8 (84%) and were on average 13.50 years.  Secondary schools from all regions of the Netherlands were randomly selected and contacted to recruit teachers in relevant school subjects. Teachers were assigned to IG or CG  **Inclusion:**  Eligibility criteria for teachers involved: (i) teaching students in Grade 7 or 8, (ii) at a school level that prepares for at least higher vocational education, and (iii) willingness to adhere to the study protocol with respect to the timing of lessons.  **Exclusion** (reasons listed):  Not reported | **Intervention(s):**  The experimental curriculum ‘Multiple Choice 4 U’ was designed as 10-session classroom curricula and consisted of a student book, a video, and a teacher manual. The curriculum focused mainly on three psychosocial behavioral determinants: outcome expectancies (short-term physical, social, and other consequences and health risks), social influences (prevalence estimates, social norms, and peer pressure), and self-efficacy (risky situations, refusal, and negotiation skills, and condom use skills).  **Control:**  Regular lessons  **Sample sizes:**  IG: n= 568 students  CG: n= 539 students  In three schools, teachers from both conditions participated, in the remaining schools only one condition was represented. | | **Outcome measures of interest:**  The survey assessed students’ involvement with the behavior for (1) safe sex and psychosocial determinants of the behaviors. The psychosocial constructs pertained to knowledge (measured for safe sex) (2) attitude, (3) outcome expectancies (3) self-efficacy (4) normative beliefs from parents and friends (5) intention. Rosenberg’s (Rosenberg, 1965) self-esteem scale (baseline; 10 items, Cronbach’s a 1⁄4 0.85), a self-developed scale of attitude towards school (baseline; 16 items; Cronbach’s a 1⁄4 0.84), and evaluation statements about lessons on safe sex.  **Follow-up periods:**  The post-test was conducted within 1 month after the intervention ended and the follow-up on average 4 months after intervention ending.  **Method of analysis:**  Multilevel generalized linear models with a random intercept were used to estimate the intervention group effects. For continuous outcome measures, multilevel linear regression analyses with two levels (students nested within teachers) were used. Effect sizes (Cohen’s d) were calculated using t-test values and degrees of freedom. Binary outcome measures were analyzed with multilevel logistic regression analyses. | **Key results:**  Data for safe sex curriculum domain not reported.  Fewer experimental students than controls had a recent experience with intercourse at post-test (OR 1⁄4 0.19, CI 1⁄4 0.05 – 0.73). There were no other effects on sexual behavior items or on the composite measure of determinants at neither post-test nor follow-up. | **Limitations (author):**  (1) Attrition: 12.1% dropped out at the post-test (attrition in IG versus CG ns) and 33.0% dropped out at follow-up (attrition in IG versus CG ns). (2) Assignment to conditions was only partly random. (3) Teachers, and not schools, were assigned to conditions (risk of contamination). (4) Teachers administered the student questionnaires, which may have led students to provide desirable responses, although efforts were made to limit such bias. (5) some teachers in both conditions had taught about alcohol and/or nutrition, which could undermine the validity of the claim of transfer effects. (6) the considerably smaller number of sessions about tobacco and safe sex in the control group compared with the experimental group. (7) The relatively short duration of the study, is not optimal for examining behavior change effects. |
| **14. Ponsford, et al., (2022)** | | | | | | |
| **First author and year:**  Ponsford, R. (2022)  **Country of study:**  United Kingdom  **Aim of study:**  To assess the feasibility and acceptability of the Positive Choices program, a whole-school social-marketing intervention, in English secondary schools prior to carrying out a phase III trial of effectiveness and cost-effectiveness.  **Study design:**  Pilot Randomized Controlled Trial  **Quality assessment score:**  + | **Setting:**  Secondary schools in south-east England.  **Participants:**  6 schools, students in year 9 (13-14 years).  Of 334 schools emailed, 11 expressed interest, and eight provided consent, of which six were recruited.  **Inclusion:**  State-funded secondary schools in south-east England were eligible to participate.  **Exclusion** (reasons listed):  Not reported | **Intervention(s):**  Positive Choices was delivered for one academic year and comprised the following components: a student needs survey examining student knowledge, attitudes, and skills related to sexual health as well as their experiences of and preferences for Relationships and Sex Education lessons to enable each school to tailor the intervention to local priorities.  **Control:**  Existing sexual health-related provision  **Sample sizes:**  IG: 4 schools (n = 971)  CG**:** 2 schools (n **=** 354)  No power calculation was performed. | | **Outcome measures of interest:**  *Acceptability;* student survey. Student awareness and acceptability of the intervention and  Student-reported coverage of topics in intervention and control schools.  **Follow-up periods:**  12-month post-intervention measurement  **Method of analysis:**  Process evaluation was informed by existing frameworks [46–48]. Fidelity was defined as 70%+ delivery of specified essential elements of each component. Assessment of acceptability drew on student survey responses and structured elements of interviews with staff involved in the implementation and focus groups with year-9 students. Qualitative data were subject to thematic-content analysis using techniques drawn from grounded theory such as in vivo and axial coding, and constant comparison. | **Key results:**  *Sex education covers well/very well*  Sexual consent:  Control: n=166 (62.2)  Intervention: n=662 (82.9)  Sexual pleasure:  Control: n=66 (24.7)  Intervention: n=480 (60.1)  Masturbation  Control: n=40 (15.0)  Intervention: n=379 (47.4)  Love:  Control: n=66 (24.7)  Intervention: n=395 (49.4)  Readiness for intimacy:  Control: n=55 (20.6)  Intervention: n=412 (51.6)  Sexual rights  Control: n=69 (25.8)  Intervention: n=431 (53.9) | **Limitations (author):**  (1) The pilot focused on feasibility and no power calculation was performed. (2) Limited data received from implementation in school meant that a rigorous assessment of overall fidelity for this school could not be made (3) There may have been some participation bias in the sample as schools were largely self-selecting. |
| **15. Van Lieshout, et al., (2017)** | | | | | | |
| **First author and year:**  Van Lieshout, S. (2017)  **Country of study:**  The Netherlands  **Aim of study:**  To measure the levels of completeness and fidelity, identify factors influencing teachers’ implementation of Long Live Love+ (LLL+), and to evaluate the students’ response.  **Study design:**  Descriptive  **Quality assessment score:**  **+** | **Setting:**  Pilot implementation of LLL+ in nine Dutch secondary schools  **Participants:**  24 classes (15 in higher general continued education, 9 in pre-university training), 60 students participated 57% girls; aged 15–17). One FGD was girls-only, one boys-only.  The teachers recruited 4–5 students from their class to participate in a focus group discussion (FGD).  **Inclusion:**  Not reported  **Exclusion** (reasons listed):  Not reported | | **Intervention(s):**  Long Live Love+ (LLL+) is an online school-based sexuality education program for adolescents aged 15–17. LLL+ covers four themes (relationships; (un)safe sex and contraception; (un)safe sex and STI; and sexual diversity) with two 45-min lessons each. Exercises are interactive and include discussions, (online) quizzes, narratives and videos.  **Control:**  No control group.  **Sample sizes:**  13 focus group discussions (3-7 students per FGD) | **Interview guide:**  The topic list for the students covered what exercises they remembered, how these had been implemented, and their opinions about these exercises.  **Follow-up periods:**  No follow-up period was conducted.  **Method of analysis:**  Thematic content analysis. | **Key results:**  **(1) Relationships:** Both boys and girls expressed to have learned about personal differences, and differences between boys and girls in what they want in relationships, while at the same time indicated to have learned that all in all, boys and girls are not so different from each other on what they perceive important. **(2) (Un)safe sex and contraception:** Participants showed more knowledge on different types of contraceptives. They also felt more confident discussing condom use and STI testing with an unwilling partner. **(3) (Un)safe sex and STI:** They showed more knowledge on STI, STI transmission, and more positive attitudes and confidence towards testing and treatment. **(4) Sexual diversity:** Students showed more understanding about and empathy towards LGBT, for example, that there is diversity among LGBT and that life can be difficult for LGBT. | **Limitations (author):**  (1) One boys-only and one girls-only focus group were formed by coincidence,. Here, students seemed more open. (2) Teachers in this study can be regarded as early adopters. It may be that these teachers have higher levels of self-efficacy and/or more positive attitudes toward sexuality education (3) Some teachers may have felt controlled in their implementation due to the research setting. (4) Not all teachers had consistently made notes in their teacher manual regarding the level of implementation and their experience with the exercises which may have biased their recall during the interviews. |
| **16. Zmyj and Wehlig, (2019)** | | | | | | |
| **First author and year:**  Zymj, N. (2019)  **Country of study:**  Germany  **Aim of study:**  To evaluate the effectiveness of a workshop against homonegativity and to identify individual characteristics that predict the effectiveness of the workshop.  **Study design:**  Quasi-experimental  **Quality assessment score:**  + | **Setting:**  A high school in a medium-sized German city  **Participants:**  A total of 214 14–16-year-old ninth graders (40% male, 56% female, 4% not specified  **Inclusion:**  Not reported  **Exclusion** (reasons listed):  Participants were excluded due to missing datasets in more than one session. | **Intervention(s):**  The workshop was part of a school curriculum organized by the teacher. It took place in the facilities of the counseling center for Lesbian, Gay, Bisexual, and Transgender (LGBT) people and lasted for about 4 h. The present workshop was developed by SchLAu NRW (Schwul-Lesbische Aufklärung Nordrhein-Westfalen, Education about gay and lesbian issues in North-Rhine Westphalia). Discussion groups, question sessions and a playful approach to breaking down gender stereotypes were constituent parts of the conception of the workshop. The face-to-face encounter plays a key role in the workshop, encouraging acceptance and respect for gay men and lesbians.  **Control:**  Waiting-list control group  **Sample sizes:**  IG =142  CG = 72  No power calculation was performed. | | **Outcome measures:**  (1) Homonegativity: was assessed by a short form of the “Attitudes Toward Lesbians and Gay Men” *Scale* (ATLG; Herek, [1988](#CIT0013); German translation: Steffens & Wagner, [2004](#CIT0032)) consisting of 10 items.  **Follow-up periods:**  Post-intervention (directly after the workshop), and follow-up (6 weeks after the workshop)  **Method of analysis:**  Linear mixed model, with group (workshop group, control group) and time point (t1, t2, t3) and group x time point interaction as fixed factors. To analyze the nature of the interaction between time and group, posthoc sample t-tests were conducted. | **Key results:**  **Homonegativity t1, mean (SD):**  IG: 1.47 (1.0)  CG: 0.82 (0.789)  **Homonegativity t2, mean (SD):**  IG: 1.10 (0.88)  CG: 0.84 (0.75)  **Homonegativity t3, mean (SD):**  IG: 1.32, (1.01)  CG: 0.89, (0.84)  Intercorrelation analysis performed for predictors of reduced homonegativity. | **Limitations (author):**  (1) The control group had lower initial levels of homonegativity and a smaller proportion of boys. (2) variables that were not specific to the workshop could have caused the change in attitude. |
